# Dynamic Covalent Boronate Chemistry Accelerates the Screening of Polymeric Gene Delivery Vectors via *In Situ* Complexation of Nucleic Acids

**DOI:** 10.1021/jacs.4c03384

**Published:** 2024-06-12

**Authors:** Bruno Delgado Gonzalez, Roi Lopez-Blanco, Samuel Parcero-Bouzas, Natalia Barreiro-Piñeiro, Lucas Garcia-Abuin, Eduardo Fernandez-Megia

**Affiliations:** † Centro Singular de Investigación en Química Biolóxica e Materiais Moleculares (CIQUS), Departamento de Química Orgánica, Universidade de Santiago de Compostela, Jenaro de la Fuente s/n, 15782 Santiago de Compostela, Spain; ‡ Centro Singular de Investigación en Química Biolóxica e Materiais Moleculares (CIQUS), Departamento de Bioquímica e Bioloxía Molecular, Universidade de Santiago de Compostela, Jenaro de la Fuente s/n, 15782 Santiago de Compostela, Spain

## Abstract

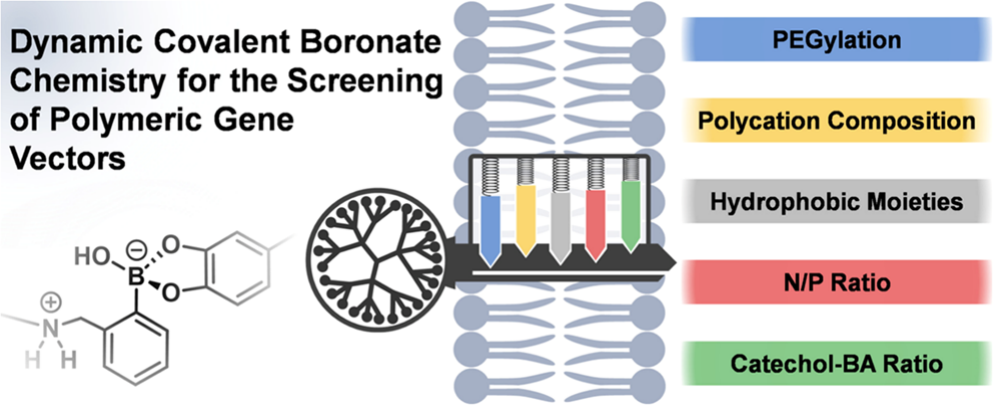

Gene therapy provides exciting new therapeutic opportunities beyond the reach of traditional treatments. Despite the tremendous progress of viral vectors, their high cost, complex manufacturing, and side effects have encouraged the development of nonviral alternatives, including cationic polymers. However, these are less efficient in overcoming cellular barriers, resulting in lower transfection efficiencies. Although the exquisite structural tunability of polymers might be envisaged as a versatile tool for improving transfection, the need to fine-tune several structural parameters represents a bottleneck in current screening technologies. By taking advantage of the fast-forming and strong boronate ester bond, an archetypal example of dynamic covalent chemistry, a highly adaptable gene delivery platform is presented, in which the polycation synthesis and pDNA complexation occur *in situ*. The robustness of the strategy entitles the simultaneous evaluation of several structural parameters at will, enabling the accelerated screening and adaptive optimization of lead polymeric vectors using dynamic covalent libraries.

## Introduction

Gene therapy provides exciting new therapeutic opportunities beyond the reach of traditional treatments.^1,2^ The key step in gene therapy is the efficient delivery of nucleic acids to target cells and tissues, which is carried out by viral and nonviral vectors.^3^ There are four basic gene therapy strategies aimed at inhibiting, adding, replacing, or editing genes.^4^ Despite the tremendous progress in the use of viral vectors, with several drugs recently gaining regulatory approval,^5^ the extremely high cost of these treatments, complex manufacturing, and severe side effects associated with the vectors^6^ have encouraged the development of more cost-effective and safer, nonviral alternatives.^7^ These are mainly based on cationic polymers, peptides, and lipids that interact with nucleic acids electrostatically, leading to various types of particles.^8,9^ Additional advantages of nonviral vectors are their ability to accommodate a large payload and to allow redosing, as demonstrated by the rollout of lipid nanoparticles for mRNA-based COVID-19 vaccines.^7^ However, compared to viruses, which have evolved to efficiently express their genes in host cells, nonviral vectors are less efficient in overcoming cellular barriers, resulting in significantly lower transfection efficiencies. This is where the exquisite structural tunability of polymers might excel. Recent advances in synthetic methodologies and click chemistry allow researchers to impart desired functions to polymeric gene carriers by assessing diverse monomer functionalities and polymer architectures.^10−12^ Still, the path is not free of obstacles as material properties need to be engineered for each nucleic acid, including the nature of the charged groups, the polymer structure and molecular weight, and the surface charge of the polyelectrolyte complex (polyplex).^12^ Also, the polycation chemical composition is usually tailored by introducing hydrophobic moieties and poly(ethylene glycol) (PEG) chains to improve the biocompatibility, cargo protection, or transfection. In addition, certain parameters must be carefully balanced. The positive charges that allow the complexation of nucleic acids, their cellular internalization and endosomal escape are also responsible for some cytotoxicity.^13^ The same applies to PEGylation that usually affords a marked decrease in cellular internalization^14^ and endosomal escape.^15^ As a result, the identification of polymers that can replace viral vectors in clinical gene therapy has proven elusive.^12^ The need to fine-tune several structural parameters represents a bottleneck of current screening processes, which has only been partially dampened by the advent of combinatorial approaches.^16−21^ Therefore, there is an urgent demand for technologies to speed up the optimization of polymeric vectors by simultaneous adjustment of multiple variables.^22^

Dynamic covalent chemistry (DCvC) represents a promising pillar of such innovative technologies. DCvC refers to chemical transformations carried out reversibly under conditions of equilibrium control.^23^ This is the case of disulfides, hydrazones, and boronate esters, which, despite being largely exploited for the engineering of stimuli-responsive materials and the controlled release of drugs,^24,25^ have found scarce application in gene therapy.^26^ Only a handful of dynamic covalent polymerizations and postfunctionalizations of polymeric gene carriers have been described so far.^27−29^ Even fewer examples have taken advantage of DCvC to prepare polymeric carriers in the presence of nucleic acids to which they complex *in situ*.^30^ These are limited to seminal reports by Aida, Li and Yang, and Ulrich on cationic polydisulfides^31,32^ and polyhydrazones^33−35^ with known ability to transfect oligonucleotides, but not plasmid DNA (pDNA). In addition, the potential of DCvC for the *in situ* optimization of polymeric gene vectors using dynamic covalent libraries has remained largely unexplored.^35^

**Figure 1:**
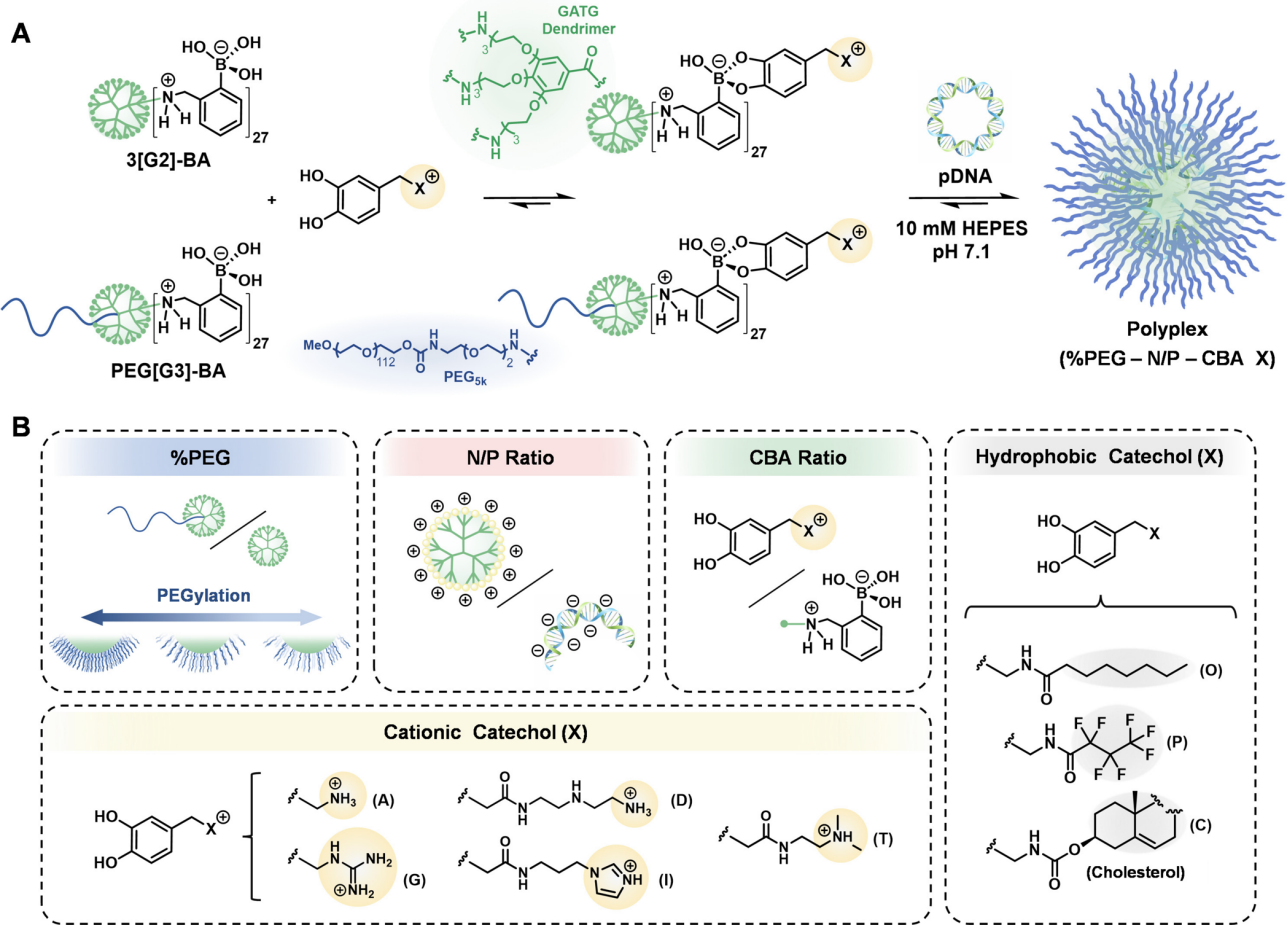
Schematic representation of the activation of polymeric boronic acid vectors and *in situ* nucleic acid complexation by the addition of cationic catechols (A). Parameters selected for simultaneous polyplex screening by dynamic covalent libraries: PEGylation degree (%PEG), N/P ratio, ratio between cationic catechols and boronic acids (CBA ratio), polycation chemical composition, and hydrophilic/hydrophobic balance (B).

Herein, we describe a highly adaptable gene delivery platform using a polymeric boronic acid that is activated in the presence of pDNA by the addition of cationic catechols (Figure 1). The fast and strong boronate ester bonding in H_2_O affords cationic dynamic polyboronates able to complex pDNA *in situ* to afford polyplexes that, without further manipulation, efficiently transfect cells. The robustness of the strategy entitles the simultaneous screening of several structural parameters at will (PEGylation, surface charge, polycation chemical composition, and hydrophilic/hydrophobic balance), enabling the adaptive optimization of lead polymeric vectors using dynamic covalent libraries.

## Results and Discussion

### Highly Adaptable Gene Delivery Platform Based on Cationic Polyboronate Vectors

Boronic acids bind diols under aqueous conditions in a pH-reversible manner, recognized as an archetypal illustration of stimuli-responsive DCvC.^36,37^ The optimal pH for boronate ester formation is above the p*K*_a_ of the boronic acid, whereas they are hydrolyzed at lower pH. These properties, along with low toxicity, have led to the widespread use of boronic acids in glycan sensing, supramolecular organizations, and sugar- and pH-responsive materials.^36,37^ To develop a polymeric boronic acid gene delivery platform, *ortho*-aminomethylphenylboronic acid (BA) was selected because of a convenient p*K*_a_*ca*. 6.5 (Figure 1).^38^ In the presence of cationic catechols, polymeric BA was expected to afford cationic polyboronates with the ability to condense nucleic acids at physiological pH while hydrolyzing upon cell internalization under acidic conditions of the endosome. A dendritic scaffold and pDNA were selected to test the feasibility of this strategy. Dendrimers are tree-like polymers with globular architecture that efficiently transfect nucleic acids when incorporating cationic peripheral groups.^39^ Their monodispersity makes them ideal candidates for evaluating new technologies and bioapplications.^40^ The selection of pDNA for *in situ* complexation was aimed to broaden the transfection scope of current cationic dynamic polymers, which are limited to oligonucleotides.^30−35^ Thus, a dendrimer and a PEG_5k_-dendritic block copolymer of the gallic acid-triethylene glycol (GATG)^41,42^ family with 27 peripheral azides were selected for functionalization with terminal BA groups, following a one-pot, three-step reductive amination procedure described in the Supporting Information (SI). 3[G2]-BA and PEG[G3]-BA (Figure 1) were obtained in very good yields and characterized with convincing evidence by ^1^H and ^13^C NMR, IR, and MALDI-TOF MS. The completion of the functionalization was easily monitored by IR (disappearance of the intense signal of azide at *ca*. 2100 cm^–1^) and ^1^H NMR (disappearance of the characteristic signals of the methylene protons in α to the azide at *ca*. 3.40 ppm that shift to *ca*. 3.00 ppm and appearance of a new aromatic signal corresponding to the proton in *ortho* to BA at *ca*. 7.40 ppm).

Five parameters were selected for simultaneous screening as a stress test of this adaptable gene delivery platform (Figure 1): (i) PEGylation degree (%PEG), the ratio between PEG[G3]-BA and 3[G2]-BA. (ii) The N/P ratio of ionizable nitrogen atoms in the polymer (equal to BA groups) to negatively charged phosphate groups in the nucleic acid. (iii) The ratio between added cationic catechols and BA groups, referred to as the CBA ratio. (iv) The polycation chemical composition among a selection of catechols functionalized with primary (A) and tertiary (T) amines, guanidine (G), ethylenediamine (D), and imidazole (I) groups with recognized ability to condense and transfect nucleic acids. While the A and T groups were taken as standard cationizable groups of widespread use in polymeric gene delivery endeavors, G, D, and I were chosen for their reported superior endosomal escape ability.^43−45^ Finally (v), the incorporation of catechols with hydrophobic moieties, such as a medium-chain fatty acid (octanoic acid, O), a fluorocarbon chain (perfluorobutanoic acid, P), and cholesterol (C), was assessed as a means to enhance the transfection efficiency by tuning the hydrophilic–hydrophobic balance.^12^ Polyplexes from cationic polyboronates were referred to as
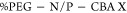
where X refers to the nature of the added catechols. For example, 7.5-5-10G refers to a polyplex prepared from a 7.5:92.5 molar ratio between PEG[G3]-BA and 3[G2]-BA, with an N/P ratio of 5 and 10 guanidinylated catechols (G) per BA group.

### Dynamic Covalent Libraries for Screening Polymeric Vectors

**Figure 2:**
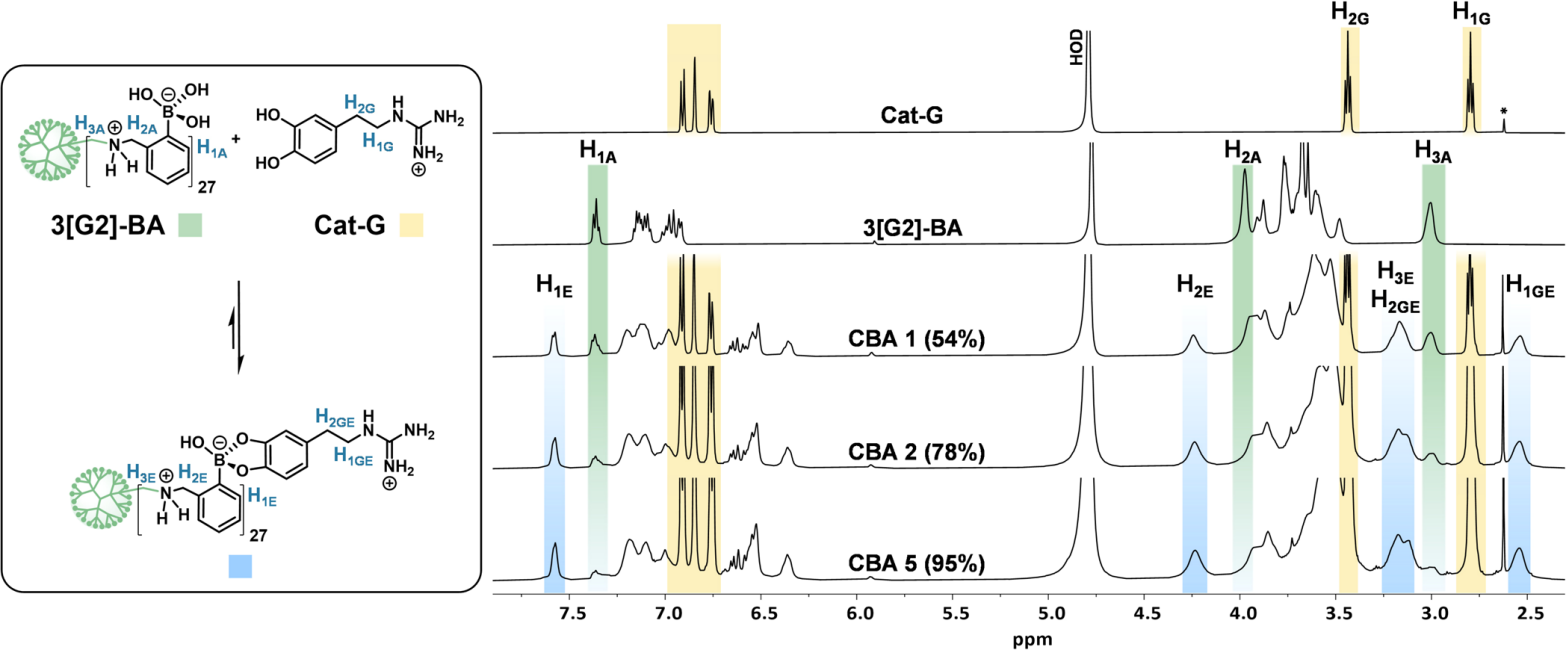
Determination of boronate ester formation by ^1^H NMR (500 MHz). Titration of 3[G2]-BA with increasing concentrations of Cat-G: Cat-G (0.62 mM in D_2_O, pH 7.1) was sequentially added to 3[G2]-BA (0.6 mL, 0.76 mM in D_2_O, pH 7.1) in an NMR tube, accounting for a molar ratio between Cat-G and BA groups (CBA) of 1, 2, and 5.

**Figure 3:**
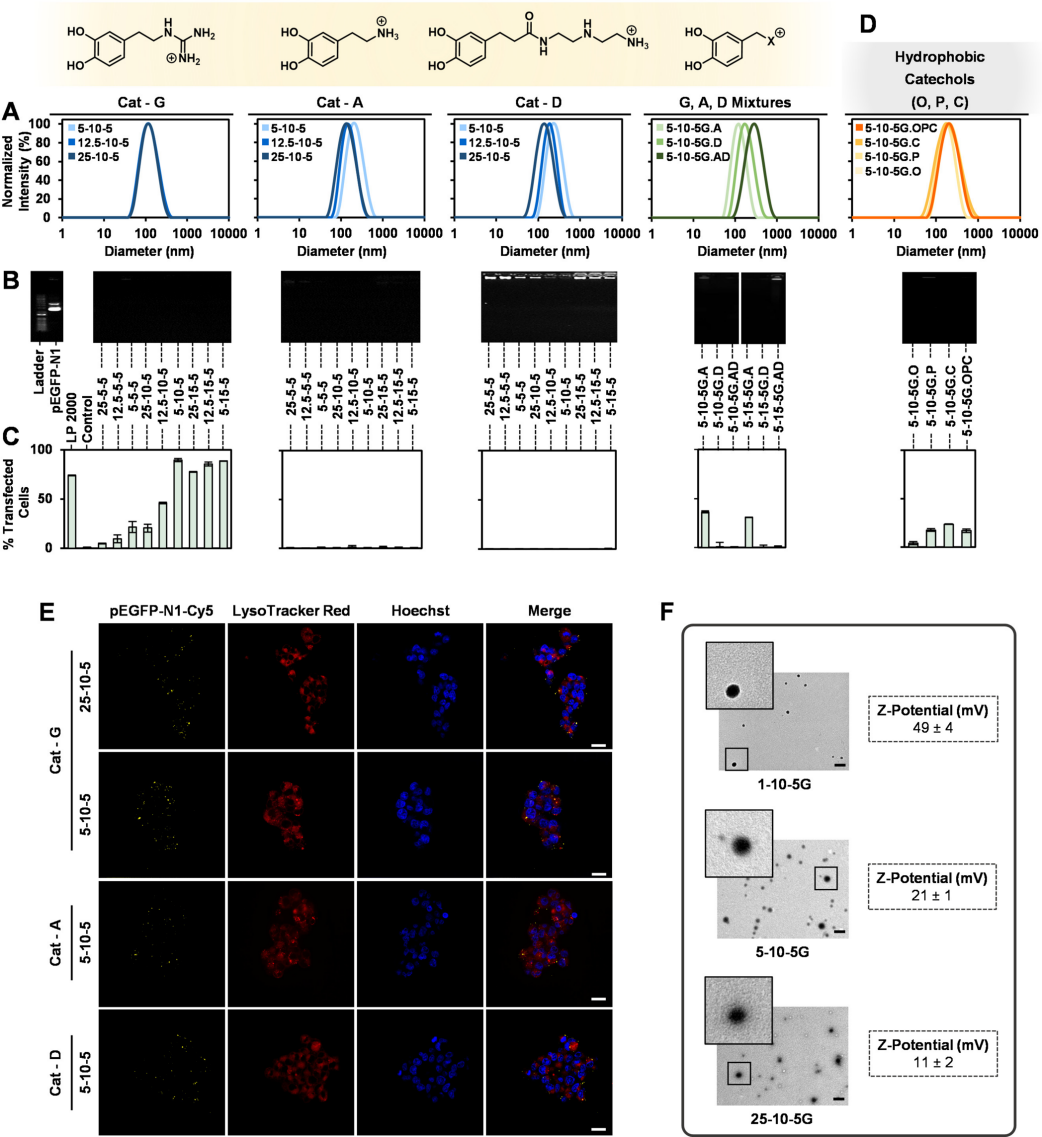
DLS size distribution of G-, A-, and D-polyplexes produced by dynamic covalent libraries (A). Evaluation of pDNA (pEGFP-N1) complexation was by gel retardation experiments with ethidium bromide on agarose gel (B). Transfection efficiency (percentage of EGFP-positive HEK-293 cells after 72 h) was measured by flow cytometry (C). Doping the 5-10-5G polyplex with 10% hydrophobic catechols (O, P, C) reduces transfection (D). LSCM images of HEK-293 cells incubated for 5 h with 25-10-5G, 5-10-5G, 5-10-5A, and 5-10-5D polyplexes show colocalization of pEGFP-N1-Cy5 (yellow) with LysoTracker Red (red), confirming their successful internalization by endocytosis. Nuclei stained in blue (Hoechst). Scale bar = 20 μm (E). TEM images and ζ potential of polyplexes with different %PEG. Scale bar = 200 nm (F).

An initial assessment of the activation of PEG[G3]-BA/3[G2]-BA and *in situ* complexation of pDNA by the addition of cationic catechols was done by dynamic light scattering (DLS) with polyplex 5-10-5G. A pDNA encoding the enhanced green fluorescent protein (EGFP) was used for these studies (pEGFP-N1). It was confirmed that neither the dendritic components nor the cationic catechol was independently capable of condensing pDNA in 10 mM HEPES, pH 7.1. Only the three-component mixture rendered polyplexes with monodisperse size distributions and a mean diameter of about 140 nm, a suitable size for gene delivery applications (Figure 3A). The pH sensitivity of the polyplexes was confirmed by DLS (Figure S1). While monodisperse size distributions were observed at pH 7.1, the polyplexes disassembled into pDNA and polymeric components at pH 5.0, evidencing hydrolysis of the boronate esters. Degradation of the vector under acidic conditions of the endosome is of relevance as it can contribute to the release and endosomal escape of pDNA and limit cytotoxicity. Before proceeding with the screening of polymeric vectors, evidence of boronate ester formation was obtained by ^1^H NMR titration of 3[G2]-BA with increasing concentrations of Cat-G (Figure 2). The progressive disappearance of the 3[G2]-BA signals centered at 7.36 (H_1A_), 3.97 (H_2A_), and 3.00 ppm (H_3A_) was accompanied by the appearance of new peaks at 7.57 (H_1E_), 4.24 (H_2E_), and 3.16 (H_3E_) ppm. In addition, new signals corresponding to Cat-G in the ester are also seen between 6.66 and 6.31 (aromatic), and at 3.16 (H_2GE_) and 2.54 (H_1GE_) ppm. Relative integration of the H_1A_ and H_1E_ protons afforded conversions of 54, 78, and 95% for the CBA ratios 1, 2, and 5, respectively. Accordingly, a CBA of 5 was set for the initial polyplexing studies. The effect of %PEG in the size of the polyplexes was also analyzed with the guanidinylated G-polyplexes of N/P 10 and CBA 5. While %PEG lower than 1% led to precipitation associated with a limited colloidal stability, values above 50% were discarded as leading to polydispersed polyplexes (Figure S2). As a result, the range of %PEG for screening was restricted to values between 1 and 25%.

Polyplexes were then produced by dynamic covalent libraries and evaluated for their size and polydispersity index (PDI), pDNA complexation (pEGFP-N1), and transfection efficiency in vitro (see the SI). These experiments were conducted in HEK-293 cells for 72 h using Lipofectamine 2000 (LP 2000) and untreated cells as positive and negative controls, respectively. A first combinatorial library was designed to get general trends about the effect of %PEG (3 different values selected: 5, 12.5, 25%), the N/P ratio (3 ratios analyzed: 5, 10, 15), and the nature of the cationic group (G, A, D, and mixtures thereof) on the polyplex performance (Figure 3). In this library, which accounted for 33 polyplexes, the CBA ratio was set to 5. DLS data for these polyplexes are shown in Figure 3A and the SI (size distributions and correlation functions in Figures S3–S13, hydrodynamic sizes and PDI in Tables S6–S10). A mean diameter of *ca*. 125–150 nm was observed for the G-polyplexes, independently of %PEG. Conversely, a size increase inversely proportional to %PEG was revealed for A- and D-polyplexes (up to 220 and 260 nm, respectively), probably associated with a lower pDNA complexing ability. No major effect of the N/P ratio on the size of the polyplexes was seen for any of the cationic catechols, independently of %PEG. Partial substitution of catechol G with various proportions of A and/or D slightly increased the polyplex size, especially for D. Despite differences in size, a complete pDNA complexation was revealed for all polyplexes by gel retardation experiments on agarose gel (Figure 3B). A decreased packing efficiency of the ethylenediamine (D) group was also evident by ethidium bromide staining of the wells on agarose gel (accessibility of the dye to polyplexed pDNA). The transfection efficiency of the polyplexes was analyzed by flow cytometry to quantify EGFP expression: the percentage of EGFP-positive cells and median fluorescence. Early experiments with polyplex 5-10-5G confirmed the necessity of the cationic catechol and dendrimers for efficient transfection (Figures S14 and S15). As seen in Figures 3C and S16, while some of the G-polyplexes showed transfection efficiencies higher than those of LP 2000, none of the A- or D-polyplexes produced any transfection, confirming a much lower performance. Similarly, the incorporation of catechols A and, particularly, D into the G-polyplexes drastically reduced transfection efficiency. Among the G-polyplexes, increased transfections were observed for the higher N/P ratios and lower %PEG. Doping one of the best-performing G-polyplexes (5-10-5G) with either alternative T or I cationic catechols (Figure S17) or catechols O, P, and C to enhance the hydrophobic interactions with pDNA (Figure 3D) did not afford improved transfections.

### Endocytosis vs Endosomal Escape of Cationic Polyboronates

While the influence of N/P on transfection efficiency was to be expected,^12^ that of %PEG could not be unequivocally associated with an impaired endocytosis or endosomal escape, since PEGylation is known to dramatically damage both steps.^14,15^ As the same picture applies to A, D, T, and I cationic catechols (low or nil transfection observed), we decided to confront this endocytosis vs endosomal escape issue by analyzing in detail the cell internalization and intracellular trafficking of some of these polyplexes using a Cy5 fluorescently labeled version of the pEGFP-N1 plasmid. Four polyplexes were selected for this study with varying %PEG and cationic catechol, namely, 25-10-5G, 5-10-5G, 5-10-5A, and 5-10-5D. Flow cytometry data of HEK-293 cells treated for 5 h with these polyplexes revealed quantitative cell internalizations and similar fluorescence intensities (Figure S18). Laser scanning confocal microscopy (LSCM) experiments performed at the same time showed for the four polyplexes a clean colocalization of Cy5 with LysoTracker Red, a well-known endosome-lysosome marker, confirming their internalization by an endocytic pathway (Figure 3E). In the absence of differences at the cell internalization step, the lower transfections for the highly PEGylated polyplexes and those incorporating cationic catechols other than G point to a deficient endosomal escape.

The physical properties of polyplexes determine their stability and performance. Given the dramatic influence of PEGylation on transfection, we decided to analyze the effect of %PEG on the morphology of the polyplexes using transmission electron microscopy (TEM). Polyplexes 1-10-5G, 5-10-5G, and 25-10-5G were selected. While TEM images for 1-10-5G showed highly compact condensates, larger %PEG resulted in particles with increasingly dense coronas (Figure 3F). Since an intimate contact between the cationic carrier and the endosomal membrane has been suggested as a requirement for efficient endosomal destabilization and nucleic acid release into the cytosol,^15,46^ such coronas might explain the poor endosomal escape of highly PEGylated polyplexes. Another polyplex parameter intrinsically related to PEGylation is the surface charge. Despite positively charged polyplexes facilitate cellular uptake and endosomal escape, more neutral particles are often preferred to avoid interaction with negatively charged serum proteins.^12^ Accordingly, the zeta potential of the above polyplexes was measured. As expected, a reduction from 49 to 11 mV was observed when increasing %PEG from 1 to 25% (Figure 3F), a charge shielding that offers great opportunities for fine-tuned gene therapy applications. Regarding the superior transfection efficiency of G-polyplexes, guanidinylated polymeric vectors have been recognized to outperform amine-derived analogs in terms of endosomal escape.^43,47,48^ Pending more conclusive studies, the net positive charge, bidentate structure, and hydrogen-bond ability of the guanidinium group^49^ emerge as a successful cocktail for nucleic acid complexation and transmembrane efficiency.^50^

### Optimizing Polymeric Vectors by Dynamic Covalent Libraries

**Figure 4:**
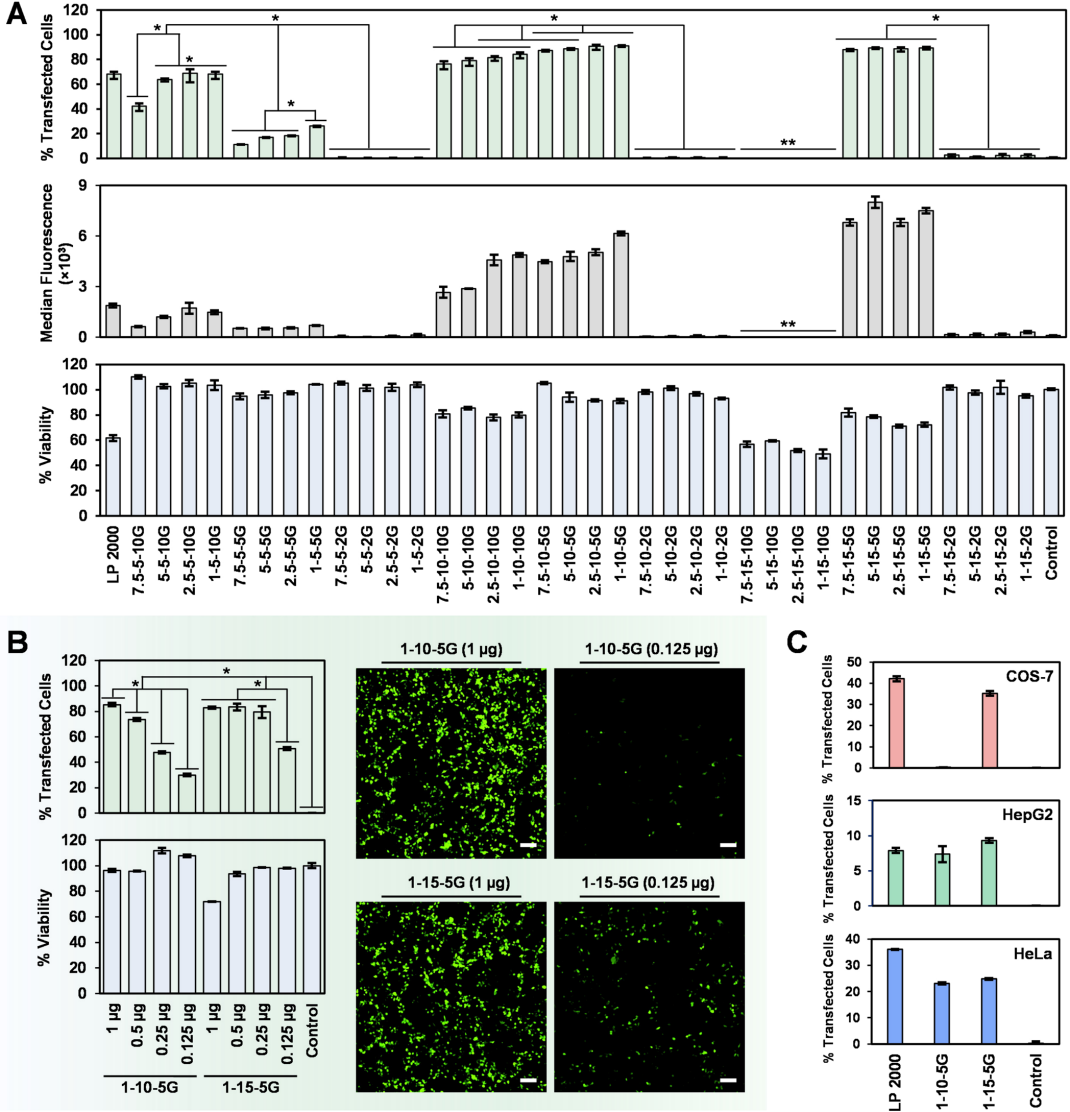
Transfection efficiency (percentage of EGFP-positive cells and median fluorescence by flow cytometry) and cell viability (CCK-8 assay) after 72 h in HEK-293 cells. (*) indicates statistical difference (*p* < 0.0001) analyzed by one-way ANOVA, followed by a Tukey multiple comparisons test. (**) did not reach the required number of events by flow cytometry (A). Dose-dependent transfection and cell viability of 1-10-5G and 1-15-5G polyplexes after 72 h in HEK-293 cells (left panel); fluorescence microscopy images of transfected cells after 72 h with different concentrations of both polyplexes. Scale bar = 100 μm (right panel) (B). Transfection efficiencies of 1-10-5G and 1-15-5G polyplexes after 48 h in COS-7, HepG2, and HeLa cells (C).

The dynamic covalent library in Figure 3 illustrates that polymeric boronic acids can be activated by cationic catechols and complex pDNA *in situ* to afford polyplexes that efficiently transfect cells. The potential of DCvC to adaptively optimize cell transfection was also demonstrated, with the best results obtained with polyplexes made of G-catechol, with %PEG lower than 12.5 and high N/P ratios. With the aim of further optimizing G-polyplexes, a second combinatorial library was planned, focusing on the screening of even lower %PEG (4 values selected: 1, 2.5, 5, and 7.5%). In this library, the N/P ratio was kept as above (same 3 values analyzed: 5, 10, 15) while the CBA ratio was varied for the first time (3 values: 2, 5, 10). A total of 36 polyplexes were produced with a size close to 150 nm (see the SI) and full pDNA complexation ability. Figure 4A shows their transfection efficiencies (EGFP expression by flow cytometry) and cytotoxicities (CCK-8 assay) in HEK-293 cells after 72 h. Transfection was highly influenced by the N/P and CBA ratios. Up to 15 formulations showed transfection efficiencies similar to or larger than LP 2000, with the highest percentage of EGFP-positive cells and median fluorescence values corresponding to N/P 10 and 15. Interestingly, the CBA ratio modulates the transfection within each N/P. Highest transfections were observed at CBA 10 for N/P 5, CBA 5 and 10 for N/P 10, and CBA 5 for N/P 15. So, a balance between both parameters is revealed, with higher CBA ratios increasing the transfection at low N/P ratios but compromising cell viability at higher N/P, especially for N/P 15. Of note, CBA 2 did not produce any transfection at all, an effect probably associated with a deficient dendrimer cationization (boronate formation, as shown in Figure 2), leading to nonfunctional carriers. As for %PEG, although differences in transfection within the 1–7.5% range are small, marginal increases are observed at lower %PEG, as already seen in Figure 3.

Among the optimized polyplexes, 1-10-5G and 1-15-5G were selected for further transfection studies, such as dose dependence and efficiency in cell lines other than HEK-293. While 1-10-5G showed a drastic reduction of transfection at plasmid concentrations below 1 μg/well, 1-15-5G proved to be more insensitive to dilution, as further verified by fluorescence microscopy imaging (Figures 4B and S15). When these polyplexes optimized for HEK-293 were tested in cell lines, such as COS-7, HepG2, and HeLa, transfection efficiencies similar to those of LP 2000 were obtained, particularly for 1-15-5G (Figure 4C).

### Protection Imparted by Cationic Polyboronate Vectors to pDNA

**Figure 5:**
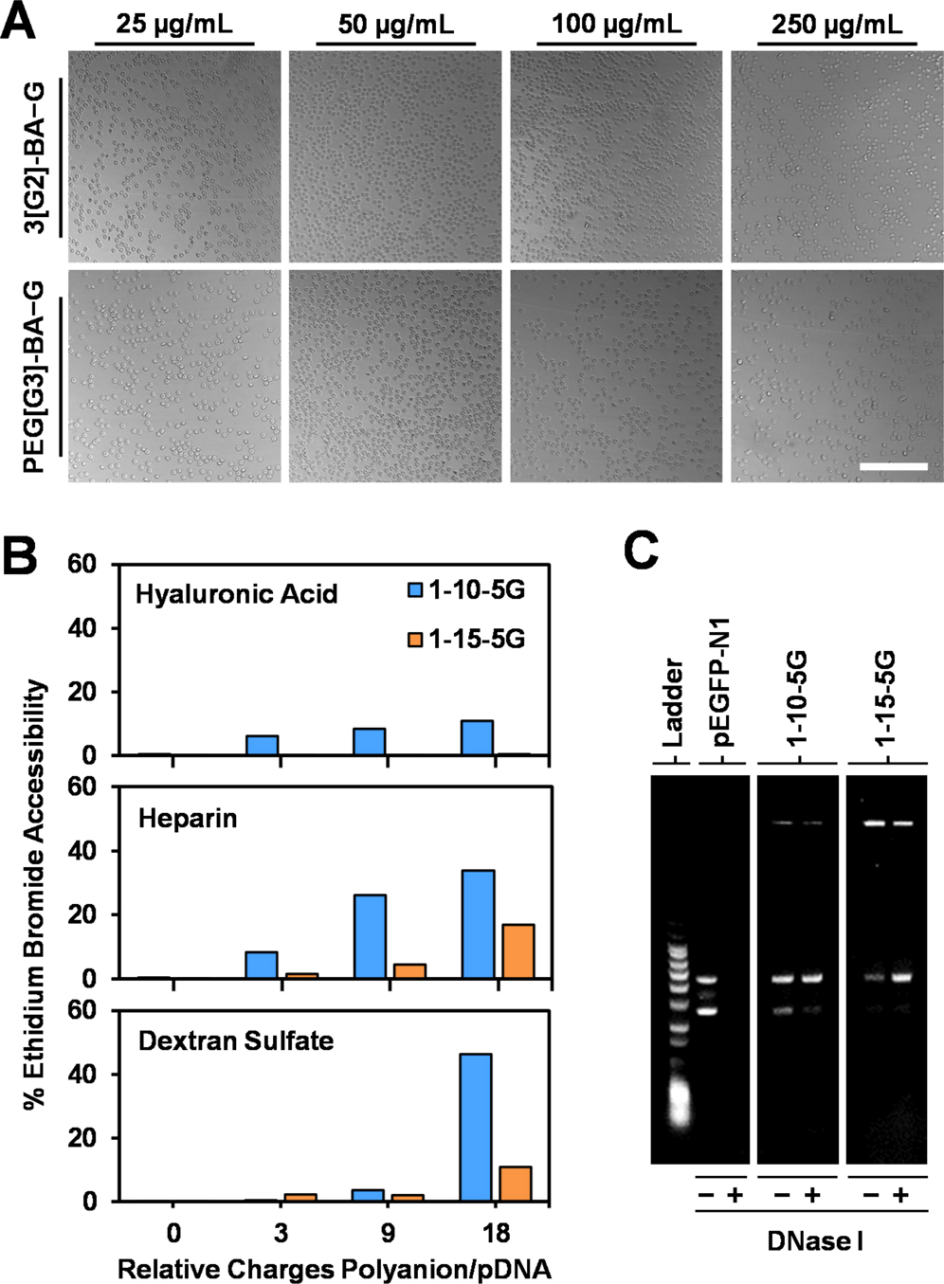
Erythrocyte aggregation test of cationic polyboronate vectors (optical microscopy). Erythrocytes (1% suspension in PBS) were incubated with increasing concentrations of 3[G2]-BA/PEG[G3]-BA and catechol G at a CBA ratio of 5 for 1 h at 37 °C. Scale bar = 100 μm (A). Protection imparted by cationic polyboronate vectors to polyplexed pDNA against decomplexation by glycosaminoglycans (measured by ethidium bromide fluorescence; 18 relative charges of polyanion/pDNA account for 138 μg/mL hyaluronic acid, 55 μg/mL heparin, and 43 μg/mL dextran sulfate) (B) and degradation by endonucleases (agarose gel electrophoresis) (C).

Since the practical application of in vivo gene therapy often requires genes to be injected directly into the bloodstream, the interaction of polymeric vectors with blood cells deserves special attention. Cationic polymers are known to electrostatically bind the membrane of erythrocytes, leading to reduced circulation half-lives and even embolism due to aggregation.^51,52^ Consequently, the blood compatibility of cationic polyboronate vectors was assessed by studying their capacity to induce aggregation (optical microscopy) and damage the membrane (hemolysis by absorbance at 450 nm) of erythrocytes. Gratifyingly, when erythrocytes were incubated for 1 h at 37 °C with increasing concentrations of 3[G2]-BA/PEG[G3]-BA and catechol G at a CBA ratio of 5, neither aggregation (Figure 5A) nor hemolysis (Figure S19) was detected even at the highest concentrations analyzed, stressing the blood compatibility of cationic polyboronate vectors.

Negatively charged proteoglycans and glycosaminoglycans abundant within serum-rich environments can displace nucleic acids from polyplexes, compromising the success of gene therapy. This is of special relevance during cell internalization when polyplexes need to penetrate the extracellular matrix.^12,53^ To assess the protection imparted by cationic polyboronate vectors to polyplexed pDNA against decomplexation by glycosaminoglycans, two of the optimized polyplexes, 1-10-5G and 1-15-5G, were incubated with increasing concentrations of highly charged polyanionic competitors (hyaluronic acid, heparin, and dextran sulfate; 2 h, 37 °C, PBS). The extent of protection was measured by monitoring the fluorescence increase of ethidium bromide, permeating the polyplex and intercalating into accessible pDNA, relative to that of untreated polyplexes and naked pDNA (positive control). Results are summarized in Figure 5B as a function of the relative number of negative charges between the polyanionic competitors and phosphate groups in pDNA. While almost no fluorescence increase was seen for polyplexes in the absence of competitors (highly compacted pDNA), increasing the concentration of polyanions resulted in a higher accessibility of ethidium bromide to pDNA, an effect more pronounced for 1-10-5G due to its lower N/P ratio. Not only do the levels of ethidium bromide accessibility compare well with values reported in the literature^54,55^ but also gel electrophoresis revealed an absence of free pDNA for 1-15-5G treated with dextran sulfate at the end point of the study, confirming the robustness of the polyplexes.

Finally, since pDNA is rapidly degraded in the presence of serum, the protection of nucleic acids from endogenous nucleases is a critical requirement in gene therapy. The ability of cationic polyboronate vectors to protect pDNA toward endonucleases was confirmed by incubating polyplexes 1-10-5G and 1-15-5G with the enzyme DNase I (1 U/μg pEGFP-N1, 60 min, 37 °C, 10 mM HEPES pH 7.1). The integrity of the polyplexed plasmid was confirmed by agarose gel electrophoresis, whereas naked pDNA was completely degraded (Figure 5C).

## Conclusions

A highly adaptable gene delivery platform is described using a dendritic boronic acid that is activated in the presence of pDNA by the addition of cationic catechols. The resulting cationic polyboronates can complex pDNA *in situ* leading to polyplexes that efficiently transfect cells. The robustness of the strategy entitles the simultaneous screening of several structural parameters at will (PEGylation, surface charge, polycation chemical composition, and hydrophilic/hydrophobic balance), enabling the accelerated screening and adaptive optimization of lead polymeric vectors by means of dynamic covalent libraries. The strategy is envisaged to be easily adapted to polymeric gene delivery vectors with alternative architectures and even nonviral peptide- and lipid-based carriers.

## Supporting Information Available

Materials, instrumentation, experimental procedures, and characterization (PDF)
