# Supplementary material for: Dynamic Covalent Boronate Chemistry Accelerates the Screening of Polymeric Gene Delivery Vectors via In Situ Complexation of Nucleic Acids
Source: J Am Chem Soc. 2024 Jun 12;146(25):17211–9. doi: 10.1021/jacs.4c03384 (PMC11212051; doi:10.1021/jacs.4c03384)
Supplement: Supplementary file 1 — ja4c03384_si_001.pdf [file ja4c03384_si_001.pdf]

Supporting Information for:

Dynamic Covalent Boronate Chemistry Accelerates the Screening of Polymeric Gene Delivery Vectors via *In Situ* Complexation of Nucleic Acids

Bruno Delgado Gonzalez,[a] *Roi Lopez-Blanco,*[a] *Samuel Parcero-Bouzas,*[a] *Natalia Barreiro-Piñeiro,*[b] *Lucas Garcia-Abuin,*[a] *and Eduardo Fernandez-Megia**[a]

[a] Centro Singular de Investigación en Química Biolóxica e Materiais Moleculares (CIQUS), Departamento de Química Orgánica, Universidade de Santiago de Compostela, Jenaro de la Fuente s/n, 15782 Santiago de Compostela, Spain.

[b] Centro Singular de Investigación en Química Biolóxica e Materiais Moleculares (CIQUS), Departamento de Bioquímica e Bioloxía Molecular, Universidade de Santiago de Compostela, Jenaro de la Fuente s/n, 15782 Santiago de Compostela, Spain.

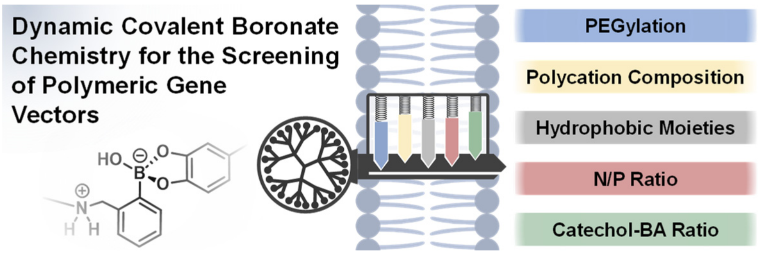

Table of Contents

1. Materials S3

2. Instrumentation S4

3. Synthesis and Characterization of New Compounds S6

4. Production of pEGFP-N1 S33

5. Polyplex Preparation and Dynamic Light Scattering Measurements S35

6. Cell Studies S50

7. *In vitro* Assays S59

8. References S61

1. Materials

Dulbecco's modified Eagle's medium (DMEM), fetal bovine serum (FBS), penicillin, streptomycin, Lipofectamine 2000 (LP 2000), and LysoTracker Red DND-99 were purchased from Invitrogen Thermo Fisher. Bisbenzimide H-33258 (Hoechst 33258) was purchased from Sigma Aldrich. Label IT Nucleic Acid Labelling Kit, Cy5 was purchased from Mirus. All other chemicals were purchased from Acros Organics, Fluka, Sigma-Aldrich, or Thermo Fisher Scientific, unless otherwise noted. All solvents were HPLC grade, purchased from Scharlab, or Sigma-Aldrich. DMF and CH_3CN were dried using a SPS800 solvent purification system from MBRAUN. Et_3N was dried under 4Å molecular sieves. H_2O was of Milli-Q grade obtained using a Millipore water purification system. Thin-layer chromatography (TLC) was done on silica ($60F_{254}$ from Merck) aluminum-backed plates. 1-(3,4-dihydroxyphenethyl)guanidine hydrochloride (Cat-G)[1] and 3-(2,2-dimethylbenzo[d][1,3]dioxol-5-yl)propanoic acid[2] were prepared following known procedures. PEG[G3]-N_3 and 3[G2]-N_3 were prepared following procedures previously described by our group.[3,4]

2. Instrumentation

NMR Spectroscopy. NMR spectra were recorded on Varian Mercury 300 MHz or Bruker DRX 500 MHz spectrometers. Chemical shifts (δ) are reported in ppm relative to the residual solvent peak (7.26 ppm for $CDCl_3$, 4.79 ppm for D_2O, 3.31 ppm for CD_3OD, 2.50 ppm for DMSO-d_6, 1.94 ppm for CD_3CN). MestReNova 14.2.2 software (Mestrelab Research) was used for spectral processing.

Infrared Spectroscopy. FT-IR spectra were recorded on a Perkin-Elmer Spectrum Two equipped with a UATR accessory.

Determination of pH Values. pH values were measured with a portable pH-meter (Crison PH25) connected to a glass electrode (Crison 52 09).

Dialysis and Ultrafiltration. Dialysis was performed with a 18 mm Spectra/Por 6 MWCO 1 kDa membrane tubing from SpectrumLabs. Ultrafiltration was performed on Amicon Ultra-15 Centrifugal Filter Unit MWCO 3 kDa from Sigma-Aldrich.

Zeta Potential. Zeta potential values of polyplexes were measured by laser doppler anemometry (LDA), measuring the mean electrophoretic mobility (Malvern Zetasizer Nano ZS, Malvern Instruments). Measurements were performed in 10 mM HEPES pH 7.1 using the Smoluchowski approximation.

Column Chromatography. Automated column chromatography was performed on a MPLC Teledyne ISCO CombiFlash RF 200 psi. Samples were adsorbed onto silica 40-63 μm from VWR Chemicals and loaded into solid cartridges. RediSep Rf 12 g columns refilled with silica 40-63 μm from VWR Chemicals were used.

Transmission Electron Microscopy (TEM). TEM measurements were performed on a JEOL JEM-1011 operated at 100 kV electron microscope equipped with a camera S5 MegaView G2. A drop of a solution of polyplexes (0.37 mg/mL) was settled on a TEM

carbon type-B film copper grid (Ted Pella, Inc.) and allowed to dry at room temperature for 12 h. Size of polyplexes were determined with ImageJ software (version 1.51j8) measuring the line intensity profile across the assemble. Average diameters were obtained by measuring the size of a representative number of polyplexes.

Mass Spectrometry. Mass spectra were recorder on a Bruker MicroTOF spectrometer using electrospray ionization (ESI) in negative or positive modes. Samples were dissolved in $CHCl_3$/MeOH or MeOH acidified with 0.1% formic acid, and injected via flow injection analysis (FIA) using a 1:1 MeOH/0.1% formic acid aq solution, flow 0.2 mL/min.

Fluorescence Microscopy. Fluorescence microscopy was performed using a Nikon Eclipse TiE microscope. Images were processed with ImageJ (1.51j8) software.

Laser Scanning Confocal Microscopy (LSCM). Confocal images were obtained on an Andor Dragonfly spinning disk confocal system mounted on a Nikon TiE microscope equipped with a Zyla 4.2 PLUS sCMOS digital camera (Andor, Oxford Instruments) and an OKO-lab incubator to maintain cells at 37 °C.

3. Synthesis and Characterization of New Compounds

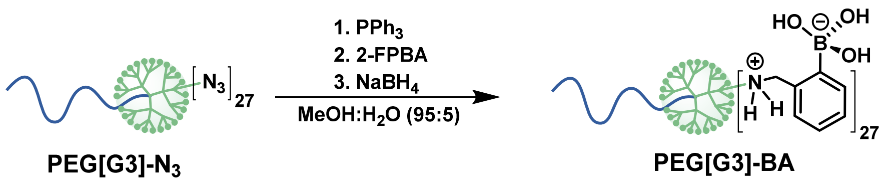

PEG[G3]-BA. PPh_3 (60.0 mg, 0.23 mmol) was added to a solution of PEG[G3]-N_3 (100.0 mg, 7.70 µmol) in MeOH:H_2O (95:5, 2.1 mL). The mixture was stirred at rt for 12 h and then, 2-formylphenylboronic acid (46.7 mg, 0.31 mmol; 2-FPBA) was added. Stirring was continued for 12 h at rt before the addition of $NaBH_4$ (11.8 mg, 0.31 mmol). After 4 h of stirring at rt, the solvent was evaporated. The crude product was dissolved in H_2O and purified by dialysis [8 × 1 L MeOH:H_2O (2:3), 8 × 1 L H_2O; MWCO 1 kDa, Spectra/Por 6]. After freeze-drying, PEG[G3]-BA (89.3 mg, 73%) was obtained as a white solid.

^{1}H NMR (500 MHz, D_2O) *δ*: 7.40-7.37 (m, 27H), 7.18-6.94 (m, 105H), 4.15-3.51 (m, ~1028H), 3.42 (s, 3H), 3.25 (br s, 2H), 3.04 (br s, 54H). ^{13}C NMR (125 MHz, D_2O) *δ*: 168.4, 151.5, 145.5, 140.9, 139.3, 128.9, 127.4, 126.9, 122.4, 106.0, 71.9, 70.9, 69.8, 69.5, 68.9, 68.1, 66.7, 66.6, 58.0, 52.4, 45.6, 39.6, 39.3. IR (ATR, cm^{-1}): 3437, 2874, 1714, 1352, 1113. MALDI-TOF MS (DHB, linear mode) *m/z*: M_p 15192, M_n 15010, M_w 15052.

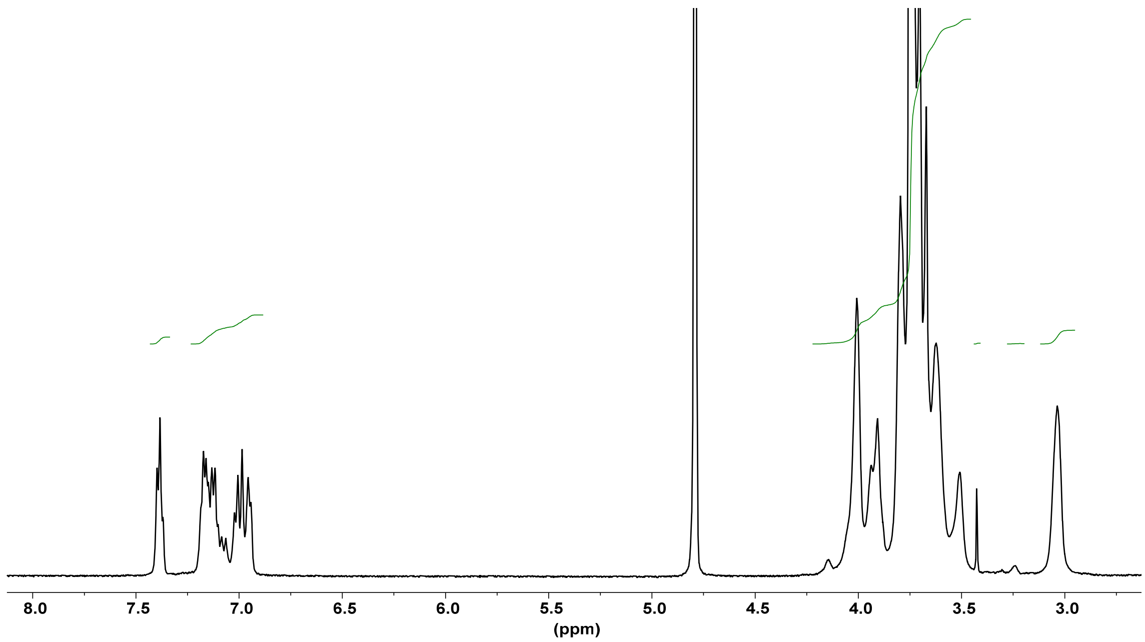

^{1}H NMR spectrum (D_2O, 500 MHz) of PEG[G3]-BA.

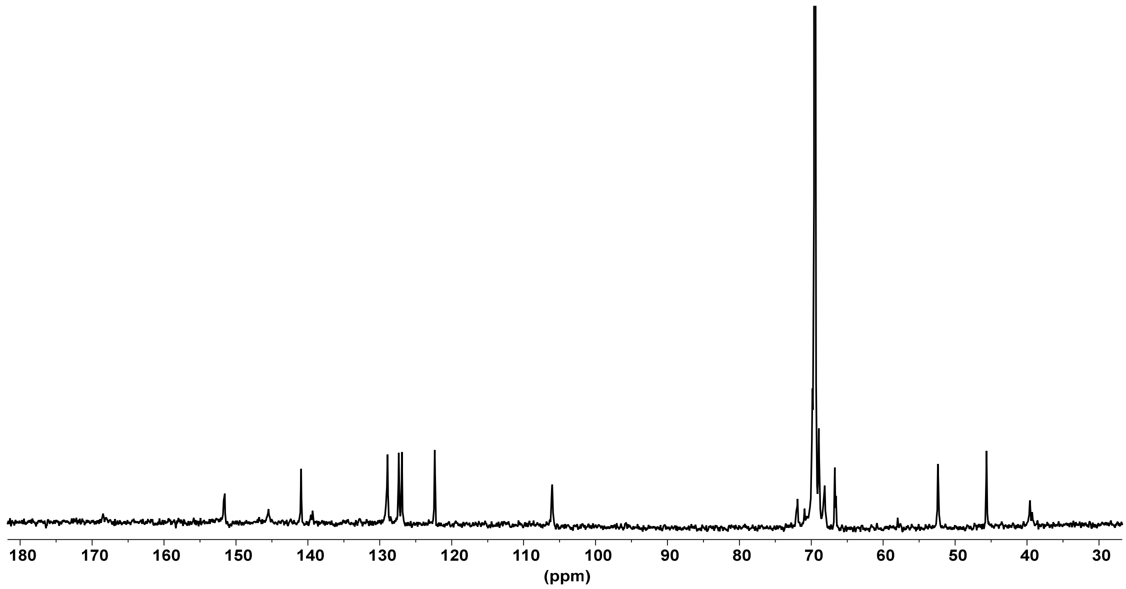

^{13}C NMR spectrum (D_2O, 125 MHz) of PEG[G3]-BA.

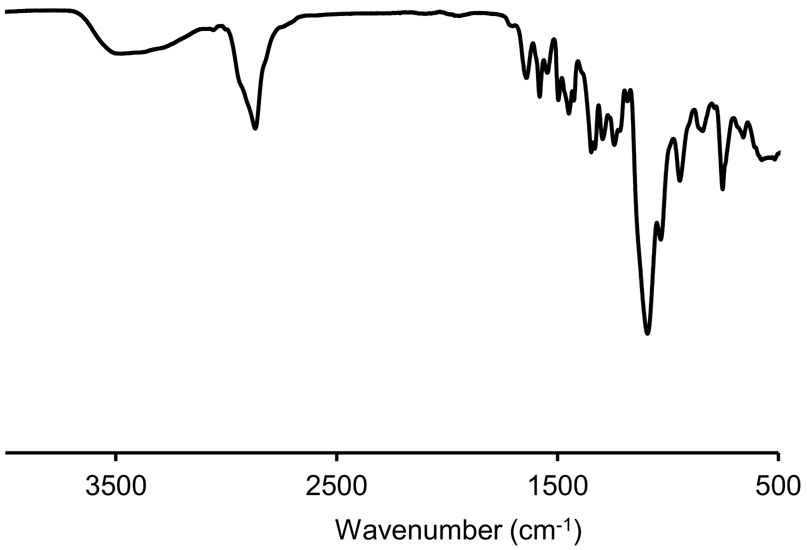

IR spectrum of PEG[G3]-BA.

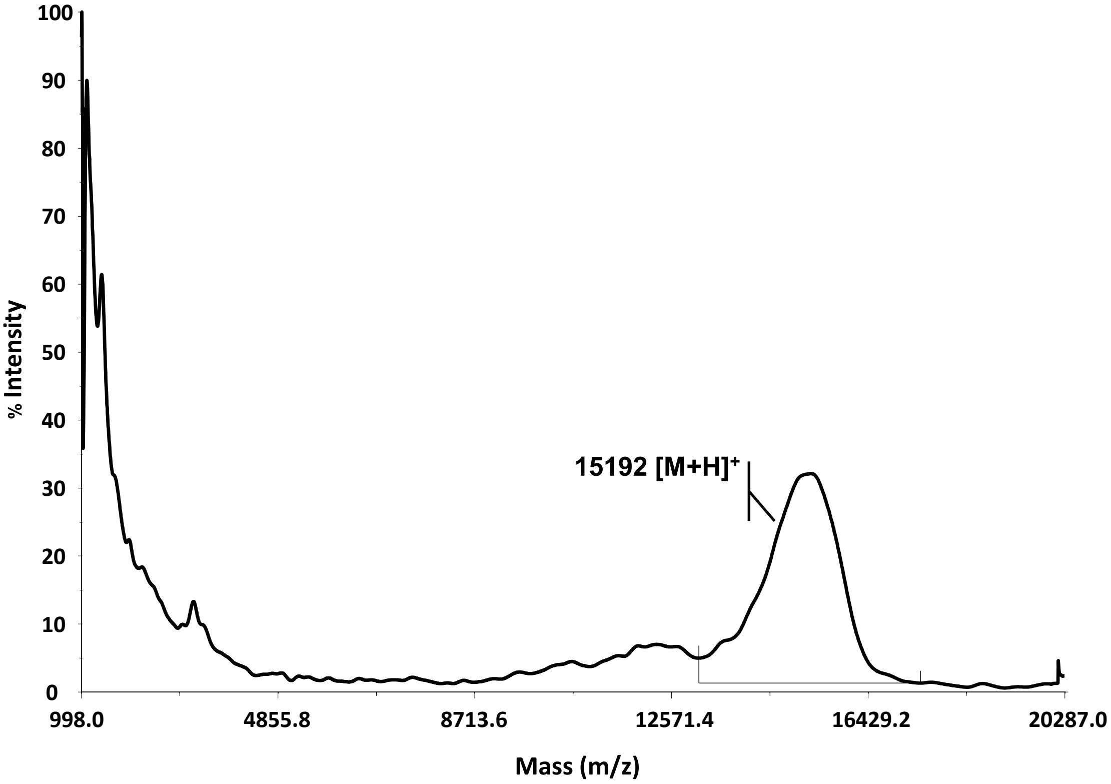

MALDI-TOF MS of PEG[G3]-BA.

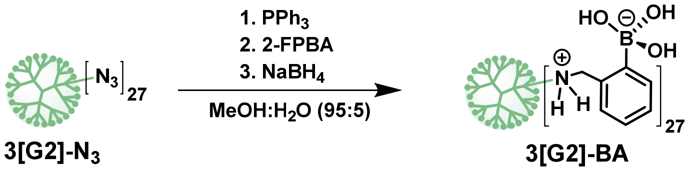

3[G2]-BA. PPh_3 (60.1 mg, 0.23 mmol) was added to a solution of 3[G2]-N_3 (60.0 mg, 7.70 µmol) in MeOH:H_2O (95:5, 2 mL). The mixture was stirred at rt for 12 h and then, 2-formylphenylboronic acid (46.8 mg, 0.31 mmol; 2-FPBA) was added. Stirring was continued for 12 h at rt before the addition of $NaBH_4$ (12.0 mg, 0.31 mmol). After 4 h of stirring at rt, the solvent was evaporated, and the crude product was dissolved in H_2O (10 mL). Then, 1 M HCl was added till pH 3. The resulting solution was washed with $CHCl_3$ (6 × 20 mL) and purified by dialysis [1 L MeOH:H_2O (2:1), 2 × 1 L H_2O; MWCO 1 kDa, Spectra/Por 6]. After freeze-drying, 3[G2]-BA (74.0 mg, 87 %) was obtained as a white solid.

^{1}H NMR (500 MHz, CD_3OD) δ: 7.41-7.36 (m, 27H), 7.10-7.08 (m, 81H), 7.08-7.01 (m, 24H), 6.02 (s, 3H), 4.15-3.91 (m, 138H), 3.83-3.41 (m, ~561H), 3.07-2.97 (m, 54H). ^{13}C NMR (125 MHz, CD_3OD) δ: 169.2, 161.5, 153.1, 142.4, 141.2, 130.7, 128.3, 127.6, 123.5, 107.4, 95.3, 73.2, 71.3, 71.1, 70.9, 70.4, 69.5, 67.8, 54.0, 47.5, 40.7. IR (ATR, cm^{-1}): 3326, 2874, 1582, 1334, 1103.

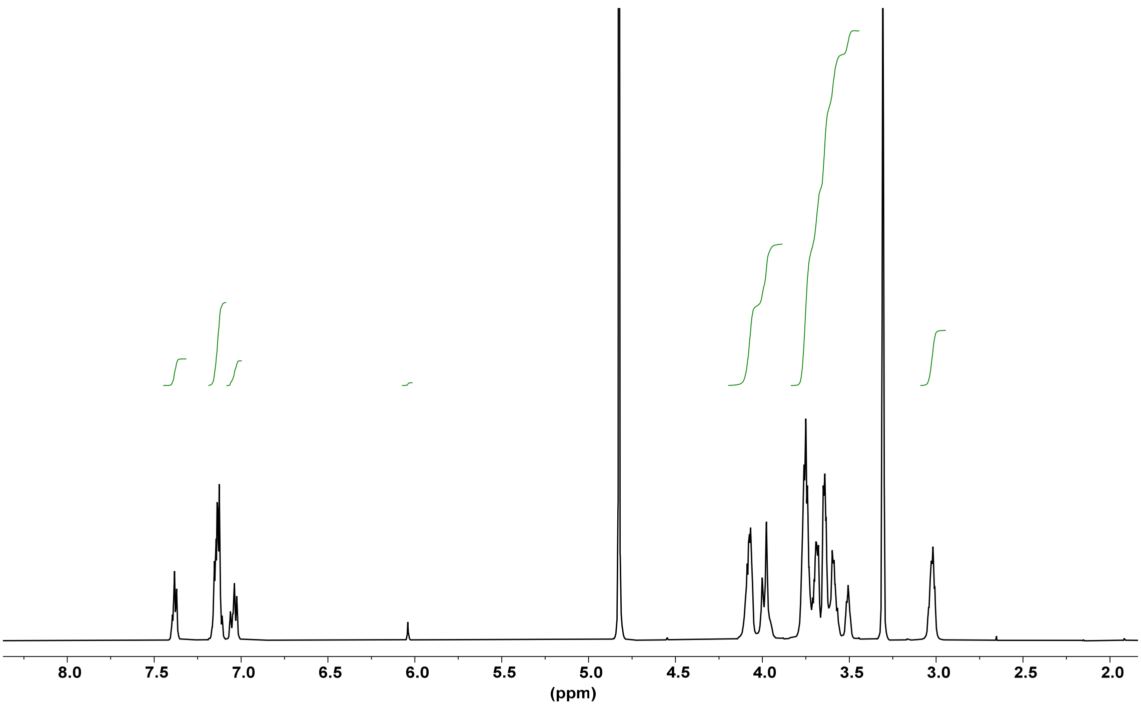

^{1}H NMR spectrum (CD_3OD, 500 MHz) of 3[G2]-BA.

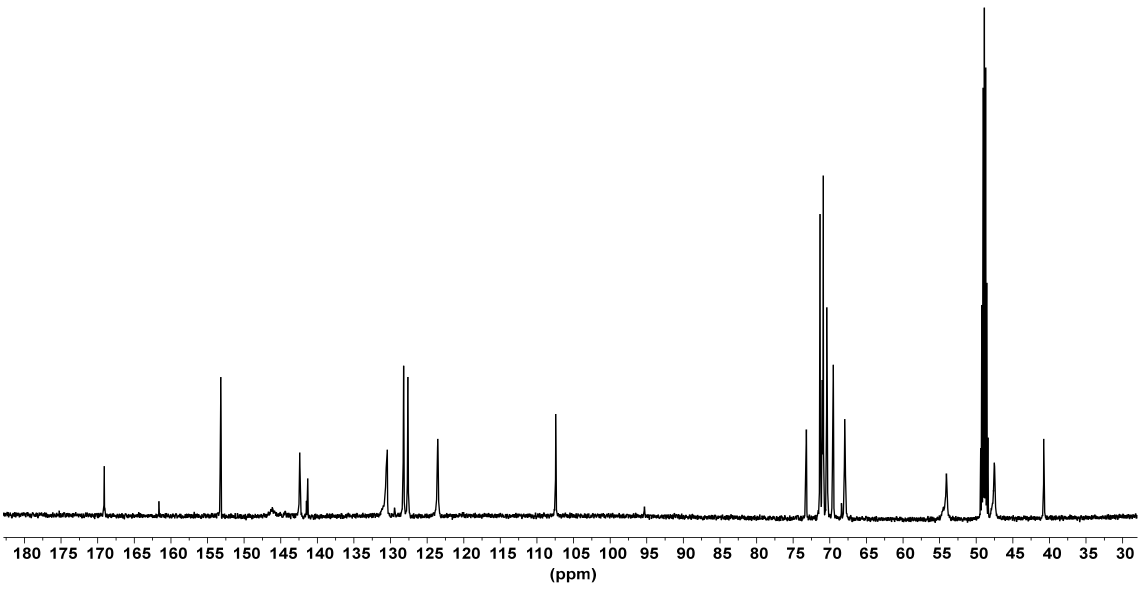

^{13}C NMR spectrum (CD_3OD, 125 MHz) of 3[G2]-BA.

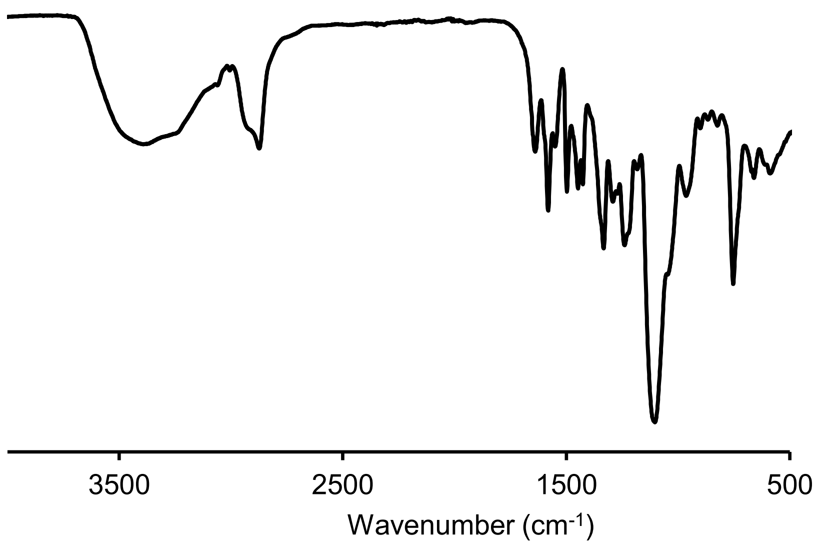

IR spectrum of 3[G2]-BA.

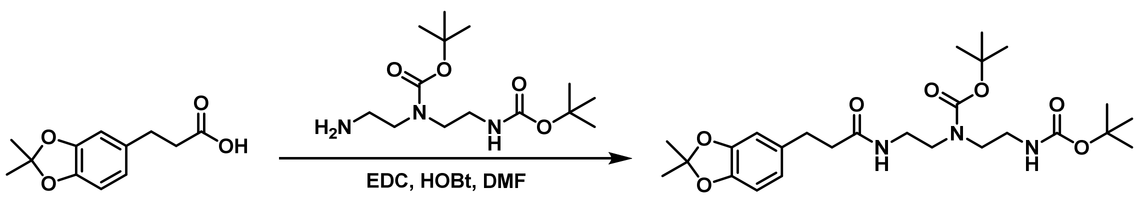

Protected Cat-D

Tert-butyl(2-((tert-butoxycarbonyl)amino)ethyl)(2-(3-(2,2-dimethylbenzo[*d*][1,3]dioxol-5-yl)propanamido)ethyl)carbamate (Protected Cat-D). HOBt (19.0 mg, 0.14 mmol), EDC·HCl (27.0 mg, 0.14 mmol), and tert-butyl (2-aminoethyl)(2-((tert-butoxycarbonyl)amino)ethyl)carbamate (43.0 mg, 0.20 mmol) were added to a solution of 3-(2,2-dimethylbenzo[d][1,3]dioxol-5-yl)propanoic acid (25.0 mg, 0.10 mmol) in anhydrous DMF (1.15 mL). After stirring overnight at rt under Ar, the reaction mixture was diluted with EtOAc (50 mL) and sequentially washed with 0.1 M HCl (3 × 50 mL), sat $NaHCO_3$ (3 × 50 mL), and brine (50 mL). The organic layer was dried ($MgSO_4$) and concentrated. The crude product was purified by automated MPLC (gradient from hexane to 50% EtOAc:hexane, silica, 15 min) to afford Protected Cat-D (30.1 mg, 65%) as a pale-yellow oil.

^{1}H NMR (500 MHz, DMSO-d_6) δ: 7.80-7.77 (m, 1H), 6.85-6.72 (m, 1H), 6.67 (td, J = 7.9, 1.7 Hz, 2H), 6.57 (dd, J = 7.9, 1.7 Hz, 1H), 3.13 (br s, 6H), 3.00 (br s, 2H), 2.68 (t, J = 6.7 Hz, 2H), 2.33-2.25 (m, 2H), 1.59 (s, 6H), 1.39 (s, 9H), 1.36 (s, 9H). ^{13}C NMR (125 MHz, CD_3CN) δ: 172.5, 171.5, 156.7, 148.2, 146.3, 135.7, 121.5, 118.6 108.9, 108.1, 80.3, 79.1, 60.9, 39.9, 38.9, 31.9, 28.5, 28.4, 25.8, 21.0, 14.4. IR (ATR, cm^{-1}): 3320, 2934, 1582, 1668, 1244, 1154. ESI-HRMS (*m/z*): 508.3004 Calcd. for $[M+H]^+$, $C_{26}H_{42}N_3O_7^+$: 508.3017.

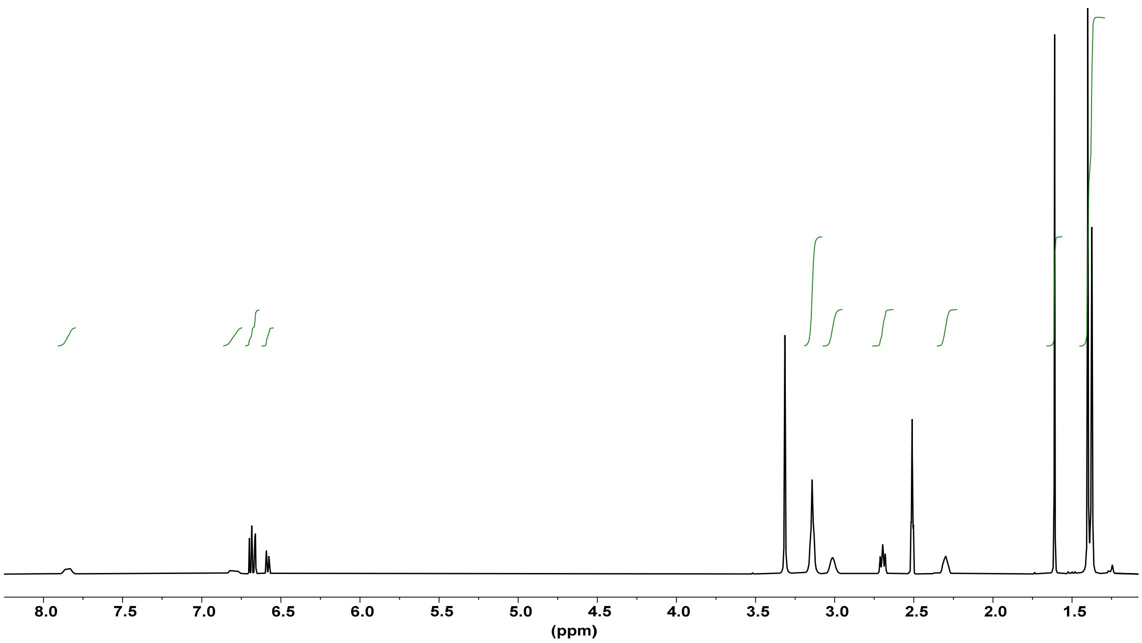

^{1}H NMR spectrum (DMSO-*d*$_6$, 500 MHz) of Protected Cat-D.

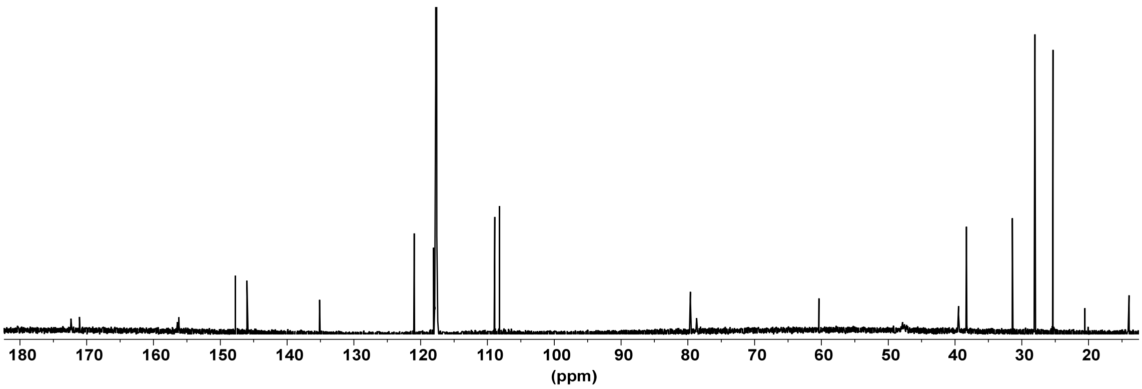

^{13}C NMR spectrum (CD_3CN, 125 MHz) of Protected Cat-D.

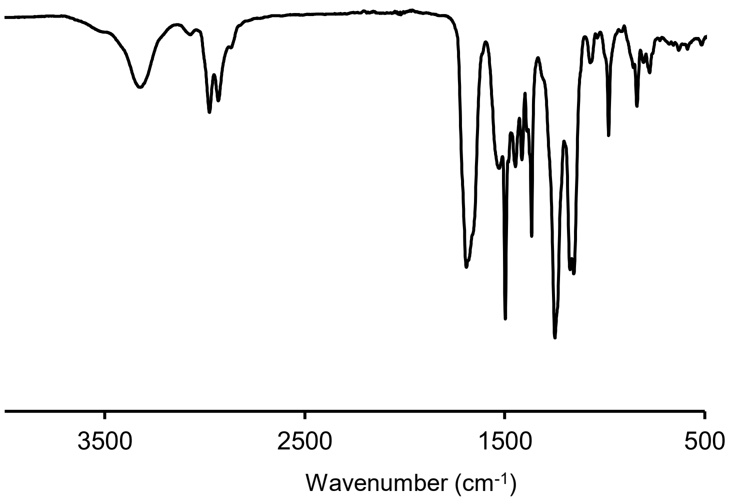

IR spectrum of Protected Cat-D.

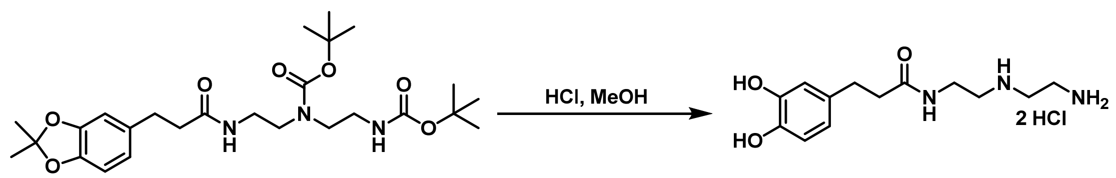

Protected Cat-D   Cat-D

***N*-(2-((2-aminoethyl)amino)ethyl)-3-(3,4-dihydroxyphenyl)propanamide hydrochloride (Cat-D).** Protected Cat-D (24.0 mg, 46.1 μmol) was dissolved in a mixture of MeOH (1.8 mL) and 37% HCl (0.45 mL). After 5 h of stirring at rt, the mixture was concentrated to afford Cat-D (13.8 mg, 88%) as a pale-yellow foam.

^{1}H NMR (500 MHz, CD_3OD) *δ*: 6.67 (td, *J* = 8.1, 2.1 Hz, 2H), 6.55 (dd, *J* = 8.1, 2.1 Hz, 1H), 3.49 (t, *J* = 5.8 Hz, 2H), 3.46-3.43 (m, 4H), 3.19 (t, *J* = 5.8 Hz, 2H), 2.78 (t, *J* = 6.9 Hz, 2H), 2.49 (t, *J* = 6.9 Hz, 2H). ^{13}C NMR (125 MHz, CD_3OD) *δ*: 177.6, 147.0, 145.4, 134.7, 121.2, 117.3, 117.1, 50.1, 46.4, 39.8, 37.8, 37.6, 32.5. IR (ATR, cm^{-1}): 3027, 1642, 1280, 1233. ESI-HRMS (*m/z*): 268.1655 Calcd. for $[M+H]^+$, $C_{13}H_{22}N_3O_3^+$: 268.1656.

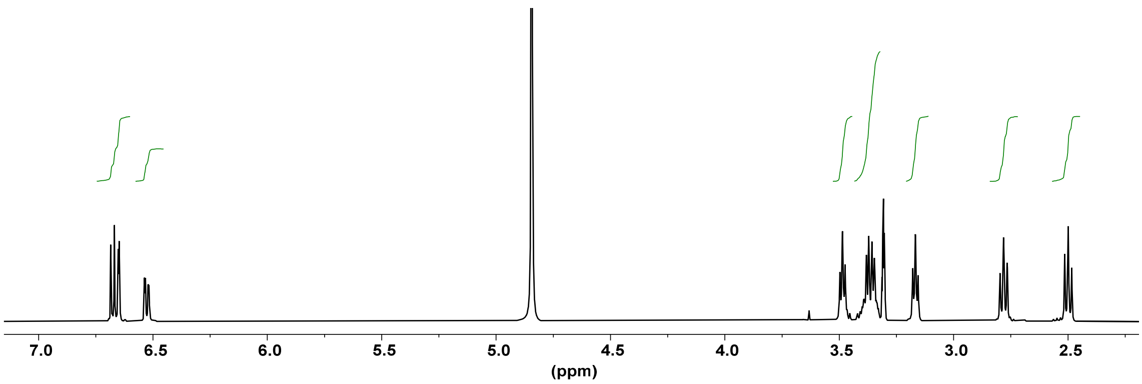

^{1}H NMR spectrum (CD3OD, 500 MHz) of Cat-D.

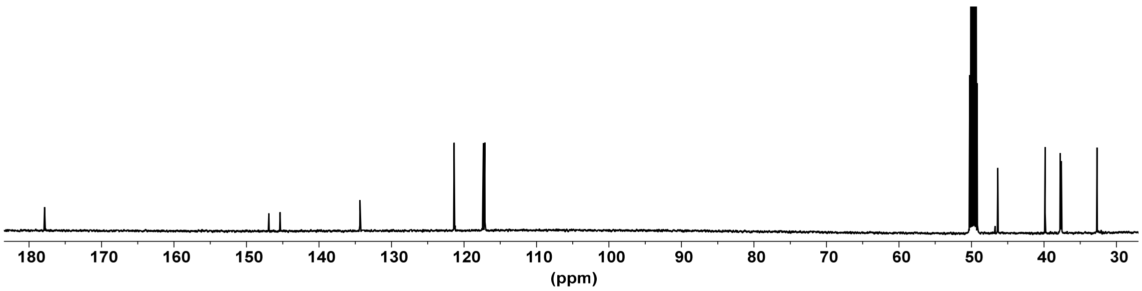

^{13}C NMR spectrum (CD3OD, 125 MHz) of Cat-D.

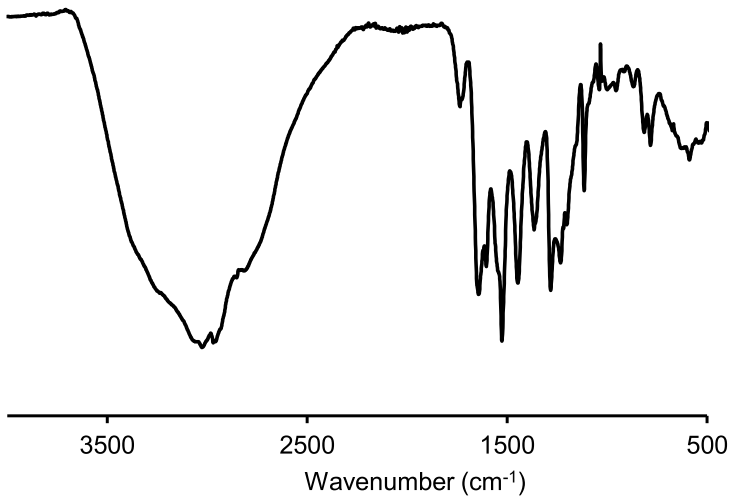

IR spectrum of Cat-D.

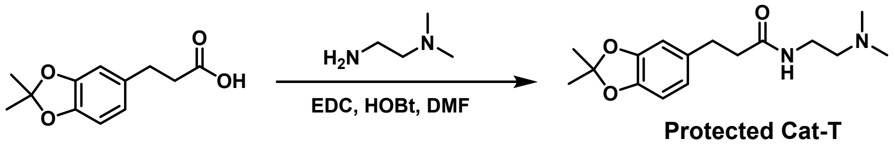

N-(2-(dimethylamino)ethyl)-3-(2,2-dimethylbenzo[*d*][1,3]dioxol-5-yl)propanamide (Protected Cat-T). HOBt (48.6 mg, 0.36 mmol), EDC·HCl (69.0 mg, 0.36 mmol), and *N*,*N*-dimethylethylenediamine (40 µL, 0.36 mmol) were added to a solution of 3-(2,2-dimethylbenzo[d][1,3]dioxol-5-yl)propanoic acid (40.0 mg, 0.18 mmol) in anhydrous DMF (1.80 mL). After stirring overnight at rt under Ar, the reaction mixture was diluted with EtOAc (50 mL) and sequentially washed with 0.1 M NaOH (3 × 50 mL) and brine (50 mL). The organic layer was dried ($MgSO_4$) and concentrated. The crude product was purified by automated MPLC (gradient from CH_2Cl_2 to 5% MeOH:CH_2Cl_2, (silica, 15 min) to afford Protected Cat-T (40.5 mg, 77%) as a pale-yellow solid.

^{1}H NMR (500 MHz, $CDCl_3$) δ: 6.62-6.53 (m, 3H), 6.23 (br s, 1H), 3.29 (q, J = 5.6 Hz, 2H), 2.83 (t, J = 7.7 Hz, 2H), 2.45-2.36 (m, 4H), 2.21 (s, 6H), 1.62 (s, 6H). ^{13}C NMR (125 MHz, $CDCl_3$) δ: 172.7, 147.7, 146.5, 134.4, 120.8, 1179, 109.0, 108.3, 58.2, 42.3, 39.0, 36.8, 31.8, 26.1. IR (ATR, cm^{-1}): 3298, 2778, 1648, 1228, 970. ESI-HRMS (m/z): 293.1871 Calcd. for $[M+H]^+$, $C_{13}H_{22}N_3O_3^+$: 293.1860.

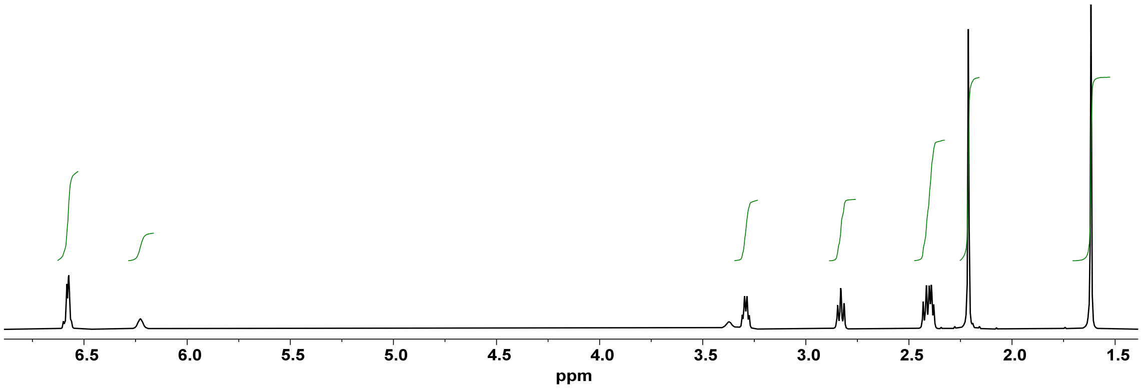

^{1}H NMR spectrum ($CDCl_3$, 500 MHz) of Protected Cat-T.

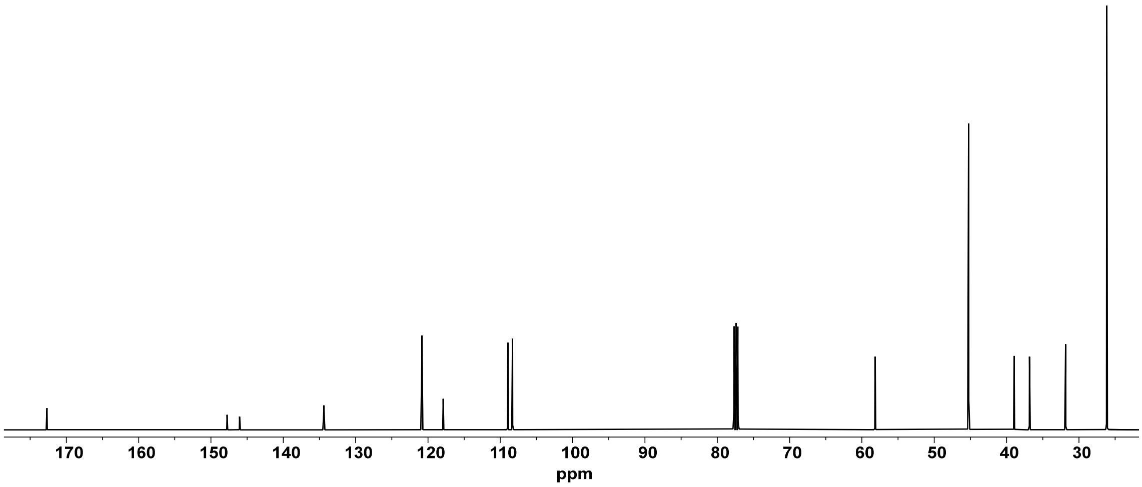

^{13}C NMR spectrum ($CDCl_3$, 125 MHz) of Protected Cat-T.

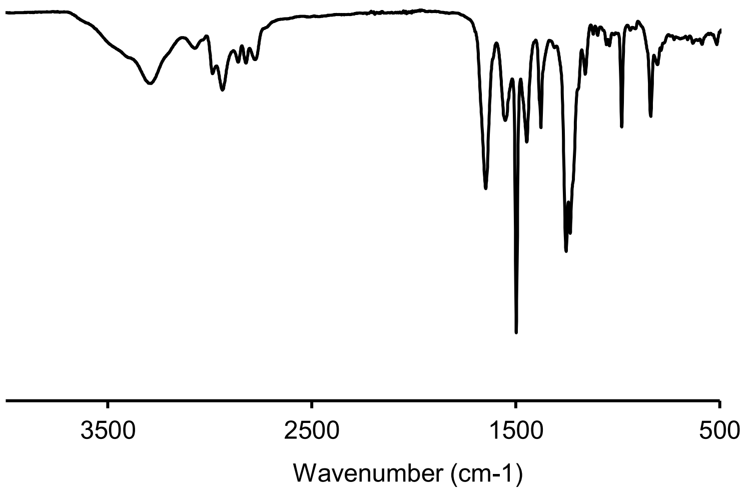

IR spectrum of Protected Cat-T.

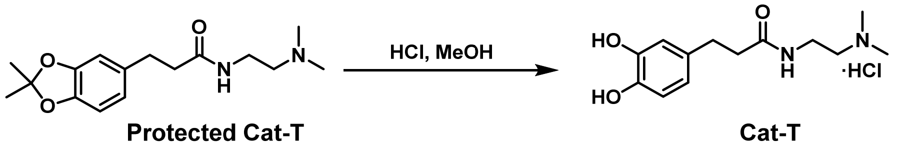

3-(3,4-dihydroxyphenyl)-*N*-(2-(dimethylamino)ethyl)propanamide hydrochloride (Cat-T). Protected Cat-T (15.0 mg, 51.1 μmol) was dissolved in a mixture of MeOH (2.24 mL) and 37% HCl (0.56 mL). After 5 h of stirring at rt, the mixture was concentrated to afford Cat-T (11.2 mg, 77%) as a pale-yellow foam.

^{1}H NMR (500 MHz, CD_3OD) *δ*: 6.72-6.61 (m, 2H), 6.53 (dd, *J* = 8.0, 2.1 Hz, 1H), 3.5 (t, *J* = 5.8 Hz, 2H), 3.16 (t, *J* = 5.9 Hz, 2H), 2.88 (s, 6H), 2.78 (t, *J* = 7.5 Hz, 2H), 2.49 (t, *J* = 7.5 Hz, 2H). ^{13}C NMR (125 MHz, CD_3OD) *δ*: 176.7, 146.2, 144.7, 135.5, 120.6, 116.6, 116.4, 58.7, 43.8, 39.0, 35.8, 31.9. IR (ATR, cm^{-1}): 3192, 2720, 1644, 1522, 1274. ESI-HRMS (*m/z*): 253.1542 Calcd. for $[M+H]^+$, $C_{13}H_{22}N_3O_3^+$: 253.1547.

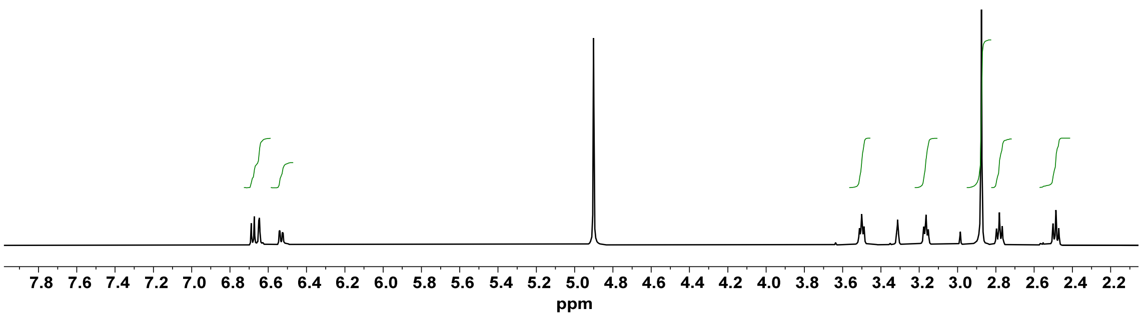

^{1}H NMR spectrum (CD_3OD, 500 MHz) of Cat-T.

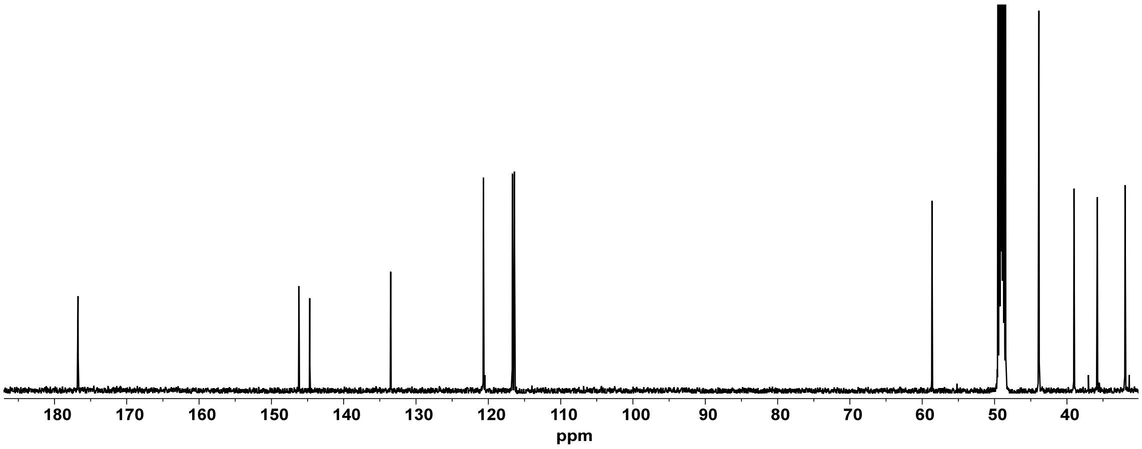

^{13}C NMR spectrum (CD_3OD, 125 MHz) of Cat-T.

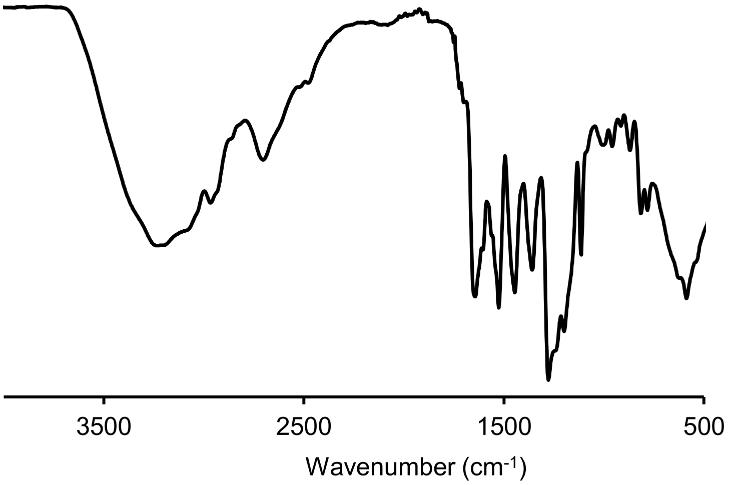

IR spectrum of Cat-T.

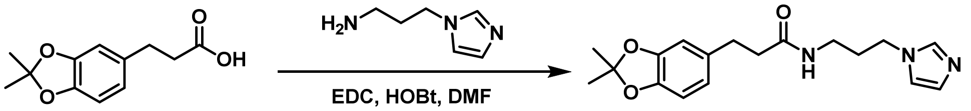

Protected Cat-I

***N*-(3-(1*H*-imidazol-1-yl)propyl)-3-(2,2-dimethylbenzo[*d*][1,3]dioxol-5-yl)propanamide (Protected Cat-I).** HOBt (48.6 mg, 0.36 mmol), EDC·HCl (69.0 mg, 0.36 mmol), and 1-(3-aminopropyl)imidazole (45.0 mg, 0.36 mmol) were added to a solution of 3-(2,2-dimethylbenzo[d][1,3]dioxol-5-yl)propanoic acid (40.0 mg, 0.18 mmol) in anhydrous DMF (1.80 mL). After stirring overnight at rt under Ar, the reaction mixture was diluted with EtOAc (50 mL) and sequentially washed with 0.1 M NaOH (3 × 50 mL) and brine (50 mL). The organic layer was dried ($MgSO_4$) and concentrate. The crude product was purified by automated MPLC (gradient from CH_2Cl_2 to 5% MeOH:CH_2Cl_2, silica, 15 min) to afford Protected Cat-I (43.9mg, 74%) as a pale-yellow solid.

^{1}H NMR (500 MHz, $CDCl_3$) *δ*: 7.40 (br s, 1H), 7.00 (br s, 1H), 6.87 (br s, 1H), 6.63-6.45 (m, 3H), 3.85 (t, *J* = 6.9 Hz, 2H), 3.17 (q, *J* = 6.4 Hz, 2H), 2.9 (br s, 1H), 2.83 (t, *J* = 7.6 Hz, 2H), 2.4 (t, *J* = 7.5 Hz, 2H), 1.9 (quint, *J* = 6.8 Hz, 2H), 1.62 (s, 6H). ^{13}C NMR (125 MHz, $CDCl_3$) *δ*: 172.8, 147.6, 145.9, 137.2, 134.0, 129.3, 120.6, 119.1, 117.8, 108.7, 108.4, 44.5, 38.6, 36.5, 31.6, 31.1, 25.9. IR (ATR, cm^{-1}): 3268, 2938, 1740, 1644, 970. ESI-HRMS (*m/z*): 330.1827 Calcd. for $[M+H]^+$, $C_{13}H_{22}N_3O_3^+$: 330.1812.

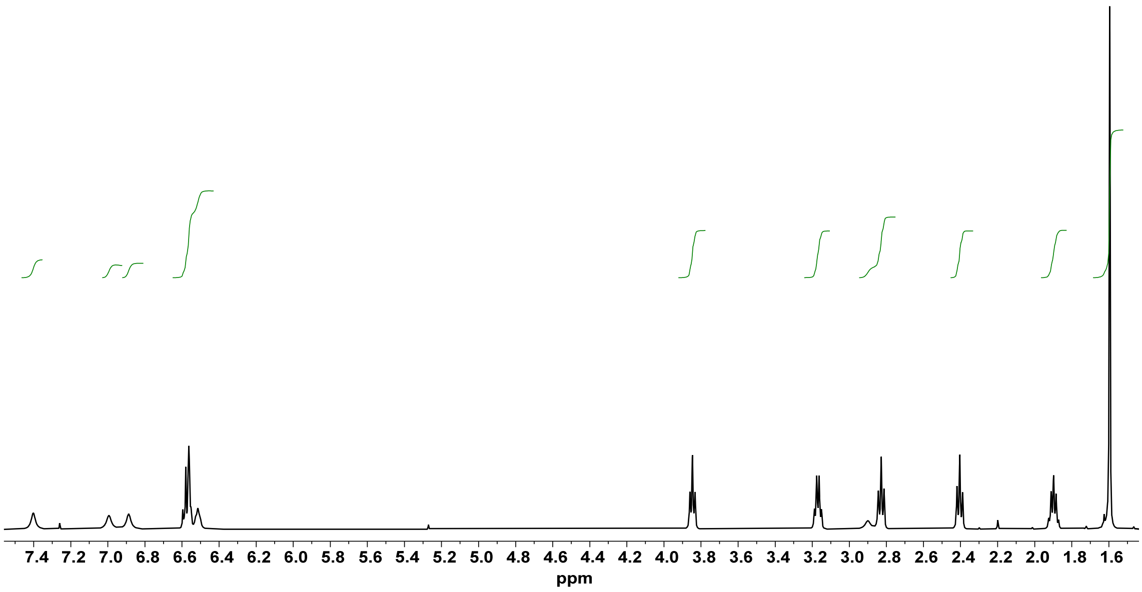

^{1}H NMR spectrum ($CDCl_3$, 500 MHz) of Protected Cat-I.

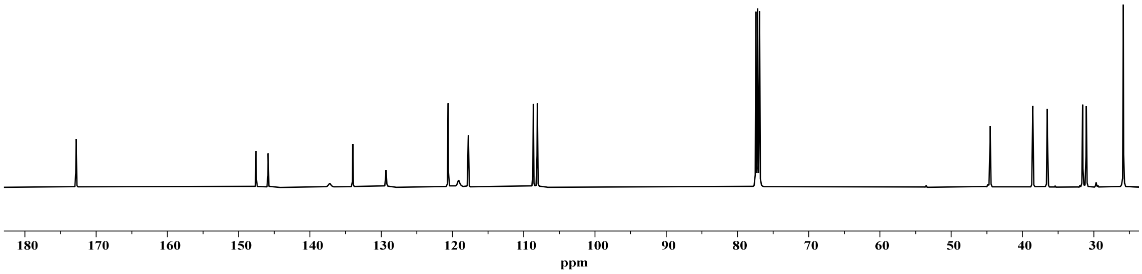

^{13}C NMR spectrum ($CDCl_3$, 125 MHz) of Protected Cat-I.

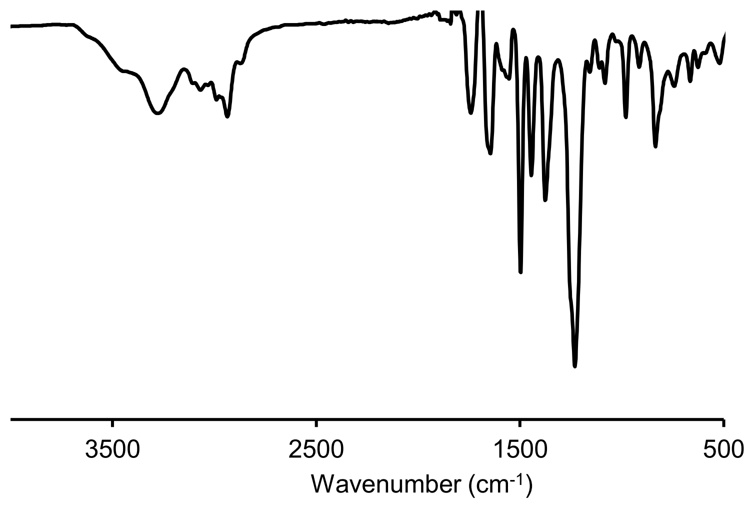

IR spectrum of Protected Cat-I.

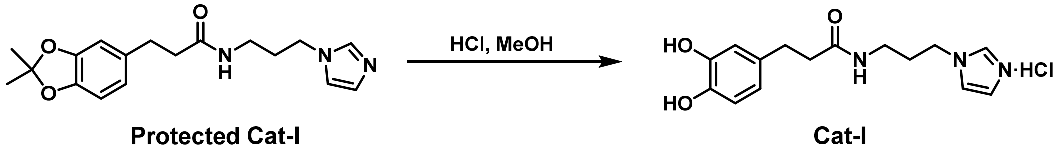

N-(3-(1H-imidazol-1-yl)propyl)-3-(3,4-dihydroxyphenyl)propanamide hydrochloride (Cat-I). Protected Cat-I (26.0 mg, 78.9 μmol) was dissolved in a mixture of MeOH (2.72 mL) and 37% HCl (0.68 mL). After 5 h of stirring at rt, the mixture was concentrated to afford Cat-I (20.2 mg, 79%) as a pale-yellow foam.

^{1}H NMR (500 MHz, CD_3OD) *δ*: 8.83 (s, 1H), 7.58 (s, 1H), 7.56 (s, 1H), 6.70-6.65 (m, 2H), 6.58-6.53 (m, 1H), 4.00 (t, J = 7.0 Hz, 2H), 3.19 (t, J = 6.3 Hz, 2H), 2.80 (t, J = 7.2 Hz, 2H), 2.51 (t, J = 7.2 Hz, 2H), 1.97 (quint, J = 6.8 Hz, 2H). ^{13}C NMR (125 MHz, CD_3OD) *δ*: 176.0, 146.1, 144.8, 136.5, 133.3, 123.3, 121.1, 120.9, 116.8, 116.4, 47.8, 38.7, 37.7, 32.1, 30.1. IR (ATR, cm^{-1}): 3140, 1640, 1520, 1283. ESI-HRMS (*m/z*): 290.1505 Calcd. for $[M+H]^+$, $C_{13}H_{22}N_3O_3^+$: 290.1499

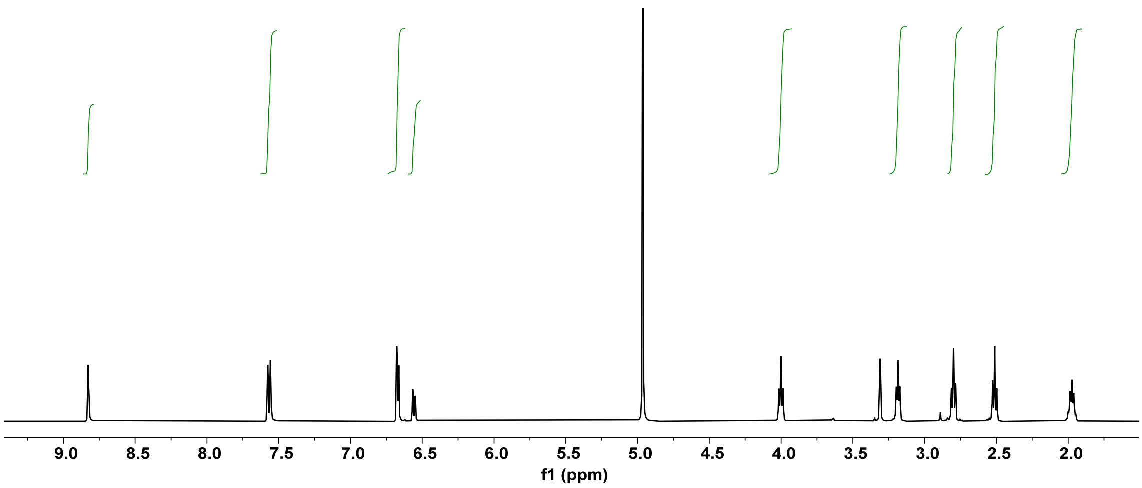

^{1}H NMR spectrum (CD_3OD, 500 MHz) of Cat-I.

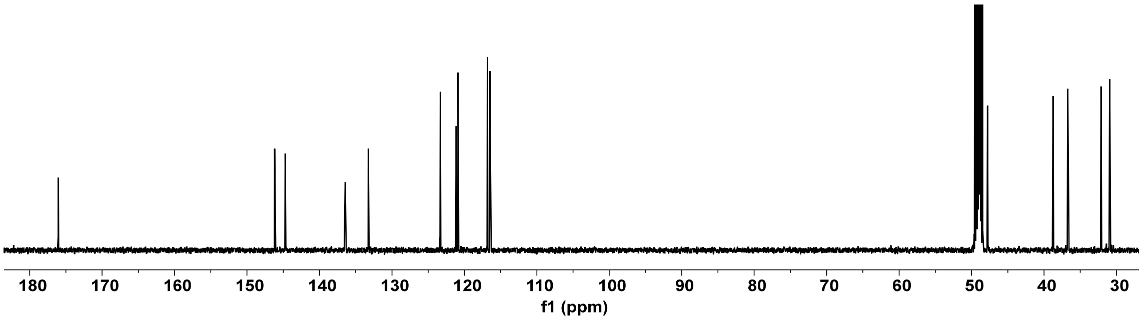

^{13}C NMR spectrum (CD_3OD, 125 MHz) of Cat-I.

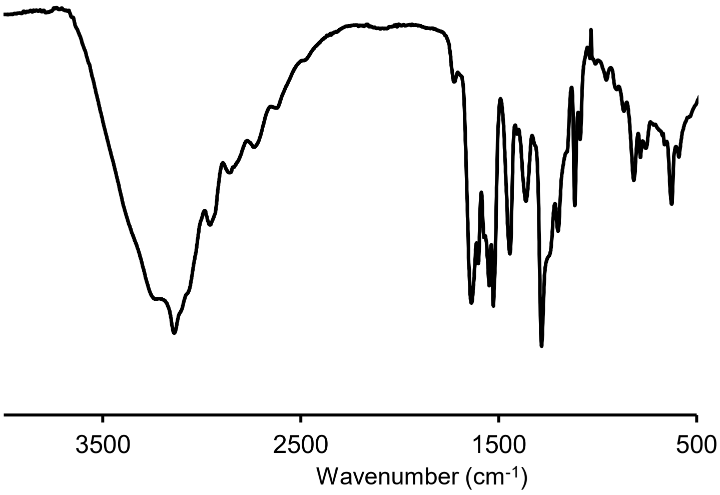

IR spectrum of Cat-I.

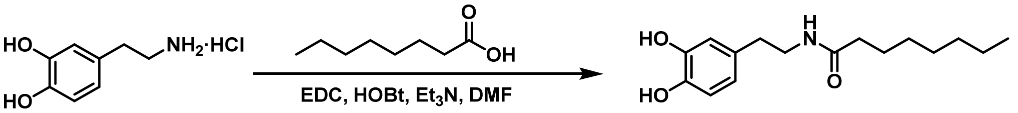

Cat-O

***N*-(3,4-dihydroxyphenethyl) octanamide (Cat-O).** HOBt (140.0 mg, 1.04 mmol), EDC·HCl (200.0 mg, 1.04 mmol), Et_3N (290 µL, 2.08 mmol), and dopamine hydrochloride (197.0 mg, 1.04 mmol) were added to a solution of octanoic acid (100.0 mg, 0.69 mmol) in anhydrous DMF (6.94 mL). After stirring overnight at rt under Ar, the reaction mixture was diluted with EtOAc (50 mL) and sequentially washed with 0.1 M HCl (3 × 50 mL), sat. $NaHCO_3$ (3 × 50 mL), and brine (50 mL). The organic layer was dried ($MgSO_4$) and concentrated. The crude product was purified by automated MPLC (gradient from hexane to 55% EtOAc:hexane, silica, 20 min) to afford Cat-O (203.8 mg, 99%) as a white solid.

^{1}H NMR (500 MHz, $CDCl_3$) *δ*: 7.82-7.61 (m, 1H), 7.44-7.31 (m, 1H), 6.85-6.69 (m, 2H), 6.55 (dd, *J* = 8.0, 2.0 Hz, 1H), 5.57 (bs, 1H), 3.47 (q, *J* = 6.7 Hz, 2H), 2.68 (t, *J* = 7.1 Hz, 2H), 2.14 (t, *J* = 7.6 Hz, 2H), 1.66-1.47 (m, 2H), 1.24 (br s, 8H), 0.86 (t, *J* = 6.5 Hz, 3H). ^{13}C NMR (125 MHz, $CDCl_3$) *δ*: 175.3, 145.0, 143.6, 130.9, 121.2, 116.1, 115.8, 41.7, 37.5, 35.4, 32.1, 30.2, 29.6, 26.5, 23.1, 14.6. IR (ATR, cm^{-1}): 3590, 1599. ESI-HRMS (*m/z*): 278.1755 Calcd. for $[M-H]^-$, $C_{16}H_{24}NO_3^-$: 278.1762.

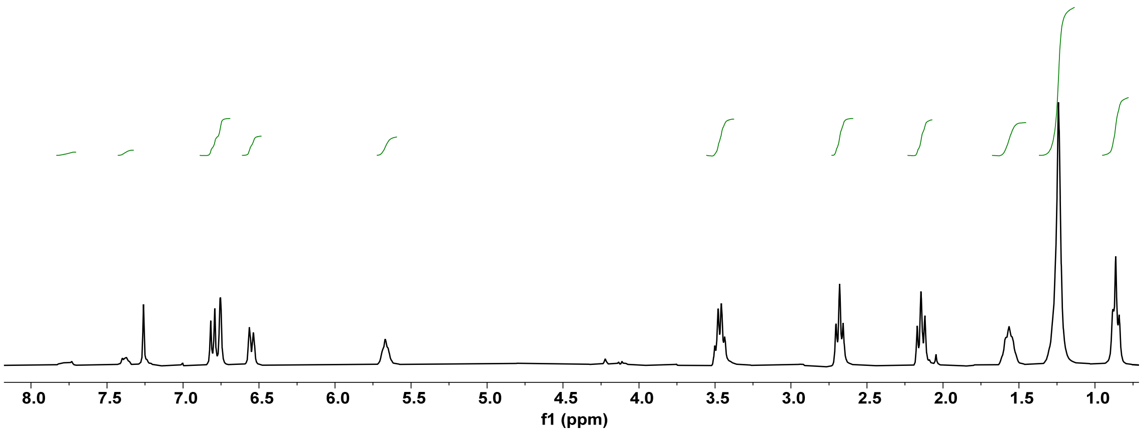

^{1}H NMR spectrum ($CDCl_3$, 500 MHz) of Cat-O.

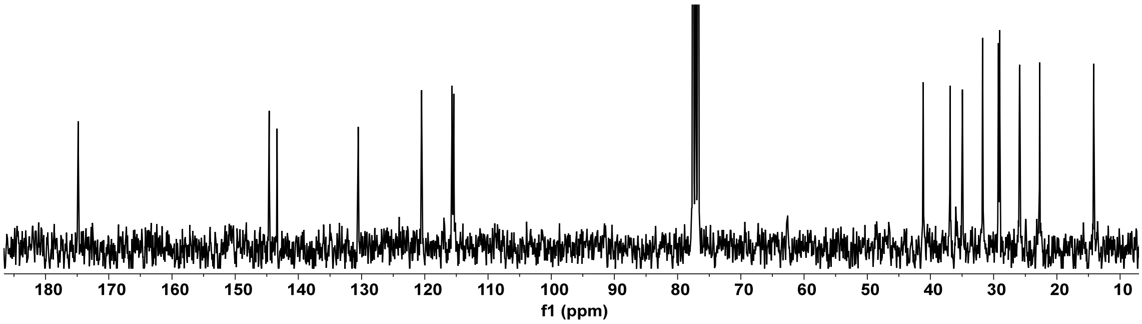

^{13}C NMR spectrum ($CDCl_3$, 125 MHz) of Cat-O.

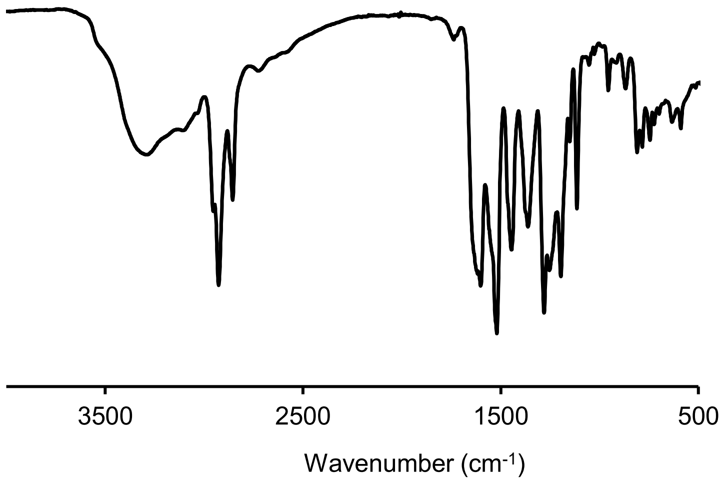

IR spectrum of Cat-O.

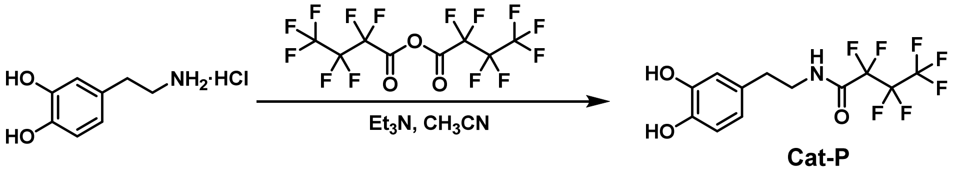

N-(3,4-dihydroxyphenethyl)-2,2,3,3,4,4,4-heptafluorobutanamide (Cat-P). Heptafluorobutyric anhydride (59.7 µL, 0.24 mmol) was added dropwise to a solution of Et_3N (100 µL, 0.73 mmol) and dopamine hydrochloride (70.0 mg, 0.37 mmol) in anhydrous CH_3CN (2.44 mL). After stirring overnight at rt under Ar, the reaction mixture was diluted with EtOAc (50 mL) and sequentially washed with 0.1 M HCl (3 × 50 mL) and brine (50 mL). The organic layer was dried ($MgSO_4$) and concentrated. The crude product was purified by automated MPLC (gradient from hexane to 40% EtOAc:hexane, silica, 15 min) to afford Cat-P (70 mg, 74%) as a white solid.

^{1}H NMR (300 MHz, CD_3OD) δ: 6.73-6.61 (m, 2H), 6.52 (d, J = 8.3 Hz, 1H), 3.45 (t, J = 7.5 Hz, 2H), 2.69 (t, J = 7.5 Hz, 2H). ^{13}C NMR (125 MHz, CD_3OD) δ: 159.1, 146.5, 145.0, 131.2, 121.1, 119.0, 117.0, 116.4, 110.0, 109.7, 42.9, 35.3. ^{19}F NMR (282 MHz, CD_3OD) δ: -86.3, -126.9, -133.5. IR (ATR, cm^{-1}): 3320, 2970, 1738, 1362, 1218, 1120. ESI-HRMS (m/z): 348.0471 Calcd. for $[M-H]^-$, $C_{12}H_9F_7NO_3^-$: 348.0476.

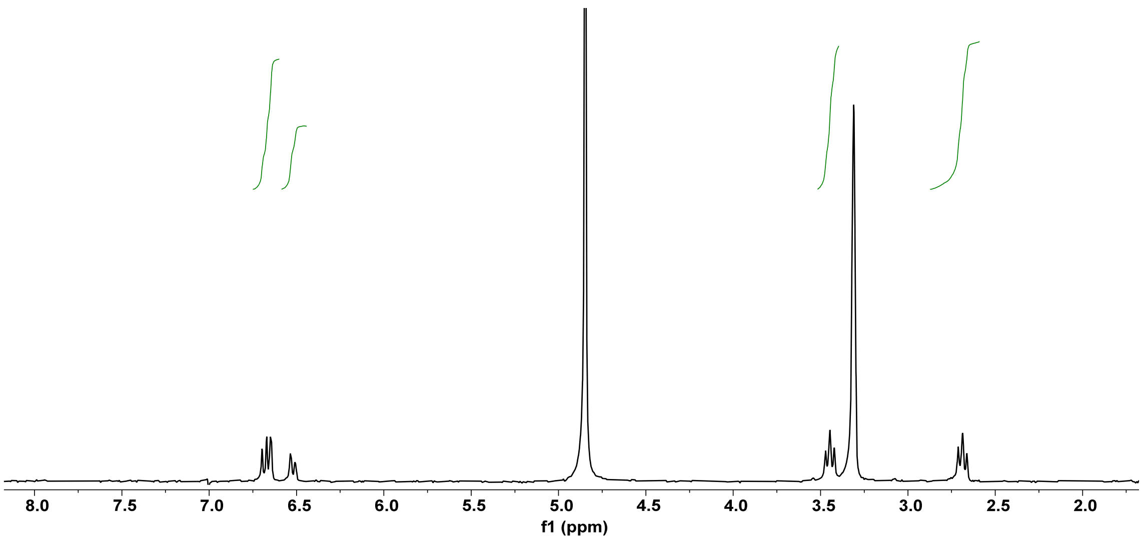

^{1}H NMR spectrum (CD_3OD, 300 MHz) of Cat-P.

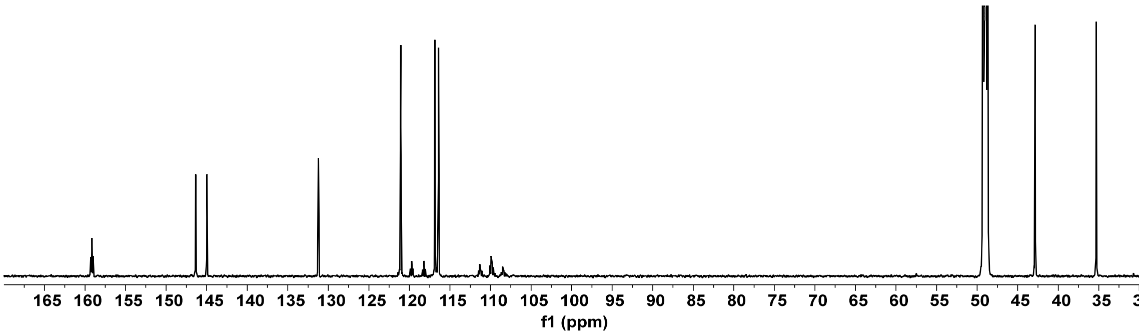

^{13}C NMR spectrum (CD_3OD, 125 MHz) of Cat-P.

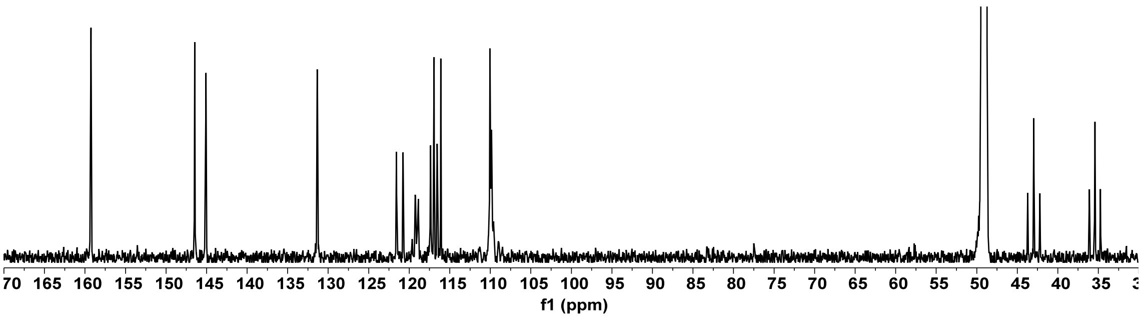

^{13}C NMR spectrum (CD_3OD, 125 MHz, ^{19}F decoupled, ^{1}H coupled) of Cat-P.

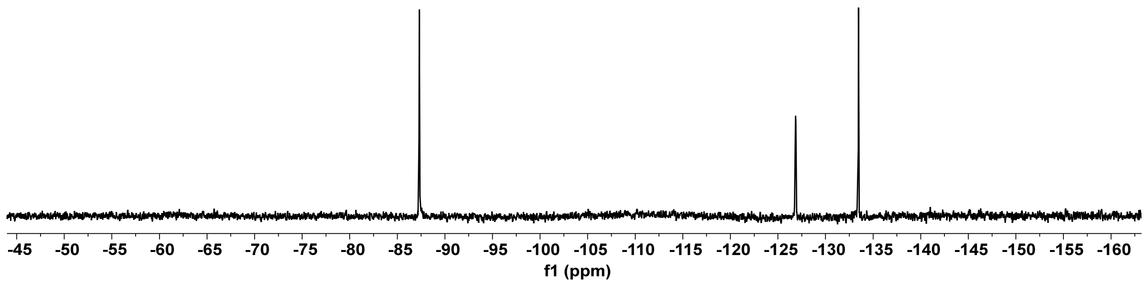

^{19}F NMR spectrum (CD_3OD, 282 MHz, ^{13}C decoupling) of Cat-P.

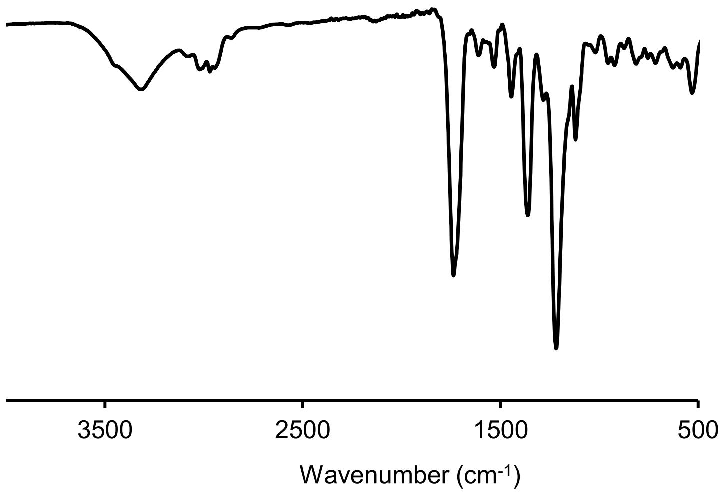

IR spectrum of Cat-P.

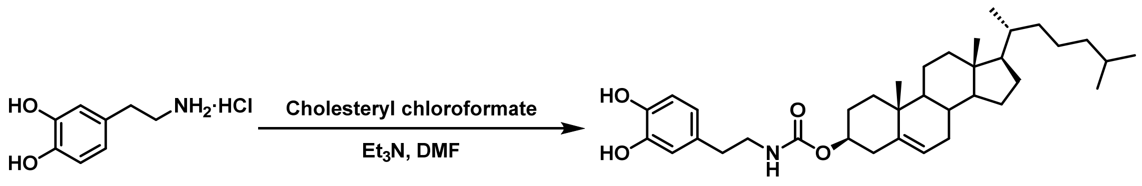

(3*S*,10*R*,13*R*,17*R*)-10,13-dimethyl-17-((*R*)-6-methylheptan-2-yl)-2,3,4,7,8,9,10,11,12,13,14,15,16,17-tetradecahydro-1*H*-cyclopenta[*a*]phenanthren-3-yl (3,4-dihydroxyphenethyl)carbamate (Cat-C). Cholesteryl chloroformate (100.0 mg, 0.22 mmol) was added to a solution of Et_3N (92 µL, 0.67 mmol) and dopamine hydrochloride (63.1 mg, 0.33 mmol) in anhydrous DMF (2.22 mL). After stirring overnight at rt under Ar, the reaction mixture was diluted with EtOAc (50 mL) and sequentially washed with 0.1 M HCl (3 × 50 mL) and brine (50 mL). The organic layer was dried ($MgSO_4$) and concentrated. The crude product was purified by automated MPLC (gradient from hexane to 40% EtOAc:hexane, silica, 20 min) to afford Cat-C (98.0 mg, 80%) as a white solid.

^{1}H NMR (500 MHz, $CDCl_3$) *δ*: 6.70 (d, *J* = 8.0 Hz, 1H), 6.62 (d, *J* = 2.0 Hz, 1H), 6.50–6.44 (m, 1H), 5.30-5.23 (m, 1H), 4.46-4.45 (br s, 1H), 3.32-3.20 (m, 2H), 2.61-2.51 (m, 2H), 2.28-2.14 (m, 2H), 2.01-1.67 (m, 5H), 1.64-1.13 (m, 14H), 1.13-0.71 (m, 23H), 0.60 (s, 3H). ^{13}C NMR (125 MHz, $CDCl_3$) *δ*: 157.7, 144.4, 143.1, 143.0, 131.4, 123.1, 121.2, 116.1, 115.8, 71.2, 57.1, 56.5, 50.3, 42.7, 42.6, 40.2, 39.9, 38.9, 37.4, 37.0, 36.6, 36.2, 35.8, 32.3, 30.1, 28.6, 28.5, 28.4, 26.3, 24.6, 24.3, 23.2, 22.9, 21.4, 19.7, 19.1, 12.3. IR (ATR, cm^{-1}): 3340, 2938, 2866, 1734, 1218, 1680, 1440, 1228. ESI-HRMS (*m/z*): 564.4052 Calcd. for $[M-H]^-$, $C_{36}H_{54}NO_4^-$: 564.4058.

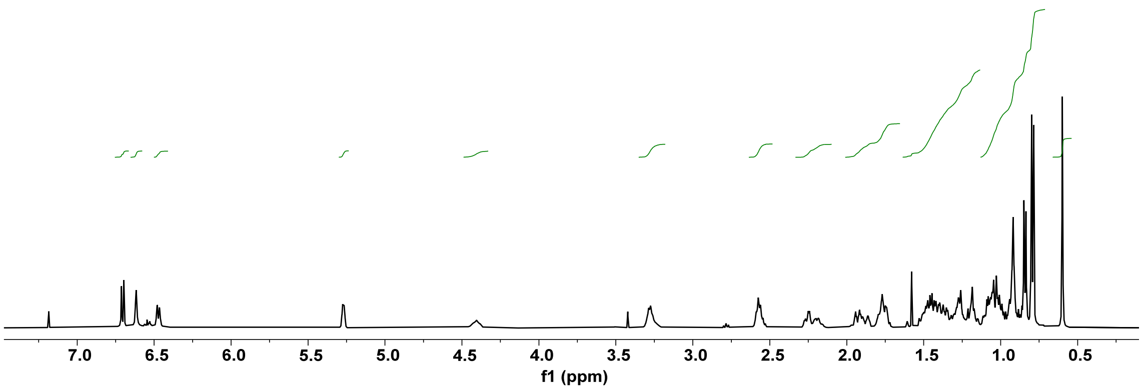

^{1}H NMR spectrum ($CDCl_3$, 500 MHz) of Cat-C.

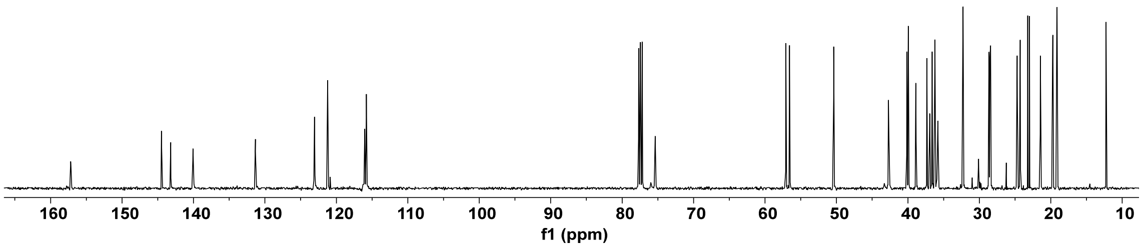

^{13}C NMR spectrum ($CDCl_3$, 125 MHz) of Cat-C.

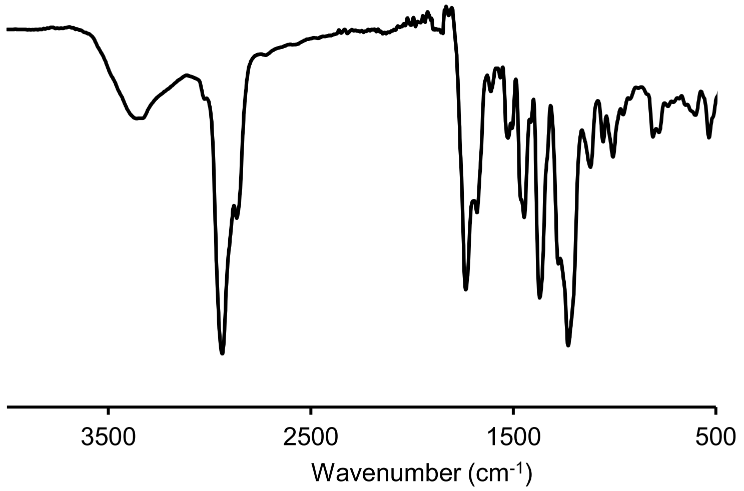

IR spectrum of Cat-C.

4. Production of pEGFP-N1

Bacteria Transformation. Competent bacteria (XL1Blue, Stratagene) were thawed on ice and a 100 μL aliquot was mixed with transforming pEGFP-N1 (1-10 ng). Following incubation on ice for 30 min, a heat shock was performed at 42 °C for 1 min. After additional incubation on ice for 2 min, 0.9 mL of LB (Luria Bertani) medium was added, and the mixture was incubated at 37 °C for 1 h under orbital stirring. Finally, bacteria were collected by centrifugation and spread onto an LB-agar plate supplemented with kanamycin (50 μg/mL).

Plasmid Production. A single colony of transformed bacteria was mixed with 50 mL of LB medium and incubated overnight at 37 ºC under orbital stirring (250 rpm).

Plasmid Purification. pEGFP-N1 was isolated from 50 mL of bacterial culture using PureYield™ Plasmid Midiprep System (Promega, Madrid, Spain) following manufacturer's instructions. After isolation, pEGFP-N1 was precipitated in a mixture of 500 mM NaOAc pH 5.5 and cold EtOH (1:20 v/v). Precipitation process was continued for at least 2 h at -20 ºC and then, samples were centrifugated at 4 ºC for 30 min (16000 *g*). The resulting pellet was resuspended in sterile H_2O and washed to remove the excess of salts by ultracentrifugation (3 × 5 mL H_2O; Amicon MWCO 10 kDa).

Cy5 Fluorescent Labelling of pEGFP-N1. Labelling was conducted using 20 μg of pEGFP-N1 in 10 mM HEPES pH 7.1 (1 μg/μL) following the manufacter's recommendations. Purification was done by precipitation in a mixture of 5 M NaCl and cold EtOH (1:20 v/v). Precipitation process was continued for 24 h at -20 ºC and then, samples were centrifugated at 4 ºC for 30 min (16000 *g*). The resulting pellet was washed at room temperature with 500 μL of 70% EtOH and centrifugated at 4 ºC for 30

min (16000 *g*). Finally, the pellet was resuspended in sterile H_2O and washed to remove the excess of salts by ultrafiltration (3 × 5 mL H_2O; Amicon MWCO 10 kDa).

5. Polyplex Preparation and Dynamic Light Scattering Measurements

Preparation of the Solutions of Components. PEG[G3]-BA and 3[G2]-BA were separately dissolved in 10 mM HEPES pH 7.1 (Table S1), and the resulting solutions were aged for 1 h at rt before polyplexing. Fresh catechol solutions were prepared in 10 mM HEPES pH 7.1 (Cat-G in Table S2; Cat-A, Cat-D, Cat-T, and Cat-I in Table S3) or DMSO (Cat-O, Cat-P, and Cat-C in Table S4) just before polyplex preparation. All solutions were filtered through sterile 0.22 μm nylon filters before polyplex formation.

Table S1. Concentration (mg/mL) of solutions of PEG[G3]-BA and 3[G2]-BA used in the preparation of polyplexes with N/P 5, 10, and 15.

	N/P 5	N/P 10	N/P 15
**PEG[G3]-BA**	1.58 (99.1 μM)	3.16 (198.2 μM)	4.74 (297.3 μM)
**3[G2]-BA**	1.10 (99.1 μM)	2.20 (198.2 μM)	3.29 (297.3 μM)

Table S2. Concentration (mg/mL) of Cat-G solutions used in the preparation of polyplexes with N/P 5, 10, and 15.

	N/P 5	N/P 10	N/P 15
**CBA 2**	2.63 (11.4 mM)	5.27 (22.8 mM)	7.90 (34.2 mM)
**CBA 5**	6.58 (28.4 mM)	13.16 (56.8 mM)	19.75 (85.2 mM)
**CBA 10**	13.16 (56.8 mM)	26.33 (113.6 mM)	39.49 (170.4 mM)

Table S3. Concentration (mg/mL) of solutions of catechols A, D, T, and I used in the preparation of polyplexes with CBA 5. These concentrations account for 28.4, 56.8, and 85.2 mM for N/P 5, 10, and 15, respectively.

	N/P 5	N/P 10	N/P 15
**Cat-A**	4.35	8.70	13.06
**Cat-D**	8.63	17.26	25.89
**Cat-T**	-	16.36	-
**Cat-I**	-	18.45	-

Table S4. Concentration (mg/mL) of solutions of catechols O, P, and C used in the preparation of G polyplexes with N/P 10 and CBA 5. A 3:1 volume ratio between Cat-G (15.8 mg/mL, 68.2 mM) and the hydrophobic catechols was used, which accounts for a 10 mol% of the latter.

Polyplex	Cat-O	Cat-P	Cat-C
**5-10-5G.O**	6.35 (22.7 mM)	-	-
**5-10-5G.P**	-	7.94 (22.7 mM)	-
**5-10-5G.C**	-	-	12.86 (22.7 mM)

Preparation of Polyplexes. For each N/P ratio and %PEG, solutions of PEG[G3]-BA and 3[G2]-BA (Table S1) were mixed in different volume ratios (Table S5) to afford a total volume of 40 μL. Then, 18.8 μL of a cationic catechol solution (Tables S2 and S3 for catechols G, A, D, T, and I; Table S4 for O, P, and C) or mixtures thereof were added according to the desired N/P and CBA ratios. A 50 μL portion of this solution was immediately added over a pEGFP-N1 (4733 bp) solution (30 μL, 20 ng/μL in 10 mM HEPES pH 7.1) and polyplex formation was left overnight at 21 ºC under orbital stirring (300 rpm) in an Eppendorf Thermomixer C.

Table S5. Volume (μL) of PEG[G3]-BA and 3[G2]-BA solutions used in the preparation of polyplexes with different %PEG.

%PEG	PEG[G3]-BA	3[G2]-BA
**100**	40	0
**50**	20	20
**25**	10	30
**12.5**	5	35
**7.5**	3	37
**5**	2	38
**2.5**	1	39
**1**	0.4	39.6

Dynamic Light Scattering of Polyplexes. DLS measurements were performed after 16-24 h of polyplexing on a Malvern Nano ZS (Malvern Instruments, U.K.), operating at 633 nm with a 173º scattering angle, at 25 ºC. DLS mean diameters were obtained from the intensity particle size distribution provided by Malvern Zetasizer Software. DLS histograms were obtained from the intensity particle size distributions. Filtering of samples before DLS measurements was avoided to prevent the removal of larger assemblies or aggregates that could obscure analysis.

Table S6. Mean hydrodynamic diameter (nm) and PDI of Cat-G polyplexes.

	N/P 5		N/P 10		N/P 15	
	**Polyplex**	Diameter (PDI)	**Polyplex**	Diameter (PDI)	**Polyplex**	Diameter (PDI)
**CBA 2**	**7.5-5-2**	144 ± 4 (0.14 ± 0.01)	**7.5-10-2**	150 ± 3 (0.12 ± 0.01)	**7.5-15-2**	142 ± 5 (0.19 ± 0.04)
	**5-5-2**	157 ± 2 (0.20 ± 0.02)	**5-10-2**	157 ± 3 (0.20 ± 0.02)	**5-15-2**	140 ± 4 (0.21 ± 0.02)
	**2.5-5-2**	167 ± 1 (0.10± 0.01)	**2.5-10-2**	167 ± 1 (0.15 ± 0.01)	**2.5-15-2**	153± 5 (0.14 ± 0.03)
	**1-5-2**	147 ± 1 (0.16 ± 0.01)	**1-10-2**	148 ± 1 (0.16 ± 0.01)	**1-15-2**	167± 3 (0.09 ± 0.01)
**CBA 5**	**25-5-5**	147 ± 5 (0.20 ± 0.01)	**25-10-5**	138 ± 5 (0.19 ± 0.01)	**25-15-5**	150± 4 (0.16 ± 0.01)
	**12.5-5-5**	124 ± 4 (0.16 ± 0.02)	**12.5-10-5**	135 ± 2 (0.20 ± 0.01)	**12.5-15-5**	146 ± 1 (0.07 ± 0.01)
	**7.5-5-5**	140 ± 3 (0.15 ± 0.02)	**7.5-10-5**	136 ± 3 (0.16 ± 0.01)	**7.5-15-5**	150 ± 2 (0.16 ± 0.01)
	**5-5-5**	128 ± 4 (0.14 ± 0.01)	**5-10-5**	148 ± 3 (0.16 ± 0.02)	**5-15-5**	137 ± 5 (0.16 ± 0.01)
	**2.5-5-5**	148 ± 3 (0.17 ± 0.02)	**2.5-10-5**	148 ± 4 (0.17 ± 0.01)	**2.5-15-5**	167 ± 3 (0.13 ± 0.01)
	**1-5-5**	152 ± 3 (0.13 ± 0.02)	**1-10-5**	143 ± 3 (0.17 ± 0.01)	**1-15-5**	141 ± 3 (0.17 ± 0.01)
**CBA 10**	**7.5-5-10**	144 ± 5 (0.22 ± 0.01)	**7.5-10-10**	159 ± 6 (0.21 ± 0.01)	**7.5-15-10**	161 ± 7 (0.04 ± 0.02)
	**5-5-10**	148 ± 3 (0.20 ± 0.01)	**5-10-10**	150 ± 2 (0.17 ± 0.01)	**5-15-10**	139 ± 2 (0.16 ± 0.02)
	**2.5-5-10**	166 ± 3 (0.18 ± 0.02)	**2.5-10-10**	164 ± 5 (0.20 ± 0.01)	**2.5-15-10**	142 ± 5 (0.19 ± 0.02)
	**1-5-10**	147 ± 4 (0.17 ± 0.01)	**1-10-10**	149 ± 2 (0.15 ± 0.01)	**1-15-10**	154 ± 4 (0.20 ± 0.02)

Table S7. Mean hydrodynamic diameter (nm) and PDI of Cat-A polyplexes.

**N/P 5**		**N/P 10**		**N/P 15**	
**Polyplex**	Diameter (PDI)	**Polyplex**	Diameter (PDI)	**Polyplex**	Diameter (PDI)
**25-5-5**	124 ± 1 (0.14 ± 0.01)	**25-10-5**	133 ± 1 (0.18 ± 0.01)	**25-15-5**	134 ± 1 (0.18 ± 0.01)
**12.5-5-5**	155 ± 3 (0.17 ± 0.02)	**12.5-10-5**	160 ± 3 (0.14 ± 0.01)	**12.5-15-5**	146 ± 1 (0.07 ± 0.01)
**5-5-5**	219 ± 3 (0.07 ± 0.01)	**5-10-5**	222 ± 6 (0.14 ± 0.01)	**5-15-5**	200 ± 3 (0.10 ± 0.02)

Table S8. Mean hydrodynamic diameter (nm) and PDI of Cat-D polyplexes.

**N/P 5**		**N/P 10**		**N/P 15**	
**Polyplex**	Diameter (PDI)	**Polyplex**	Diameter (PDI)	**Polyplex**	Diameter (PDI)
**25-5-5**	159 ± 5 (0.15 ± 0.01)	**25-10-5**	153 ± 2 (0.15 ± 0.02)	**25-15-5**	164 ± 3 (0.12 ± 0.01)
**12.5-5-5**	181 ± 3 (0.12 ± 0.02)	**12.5-10-5**	185 ± 3 (0.09 ± 0.01)	**12.5-15-5**	192 ± 3 (0.08 ± 0.02)
**5-5-5**	261 ± 3 (0.13 ± 0.02)	**5-10-5**	255 ± 3 (0.11 ± 0.01)	**5-15-5**	260 ± 5 (0.15 ± 0.01)

Table S9. Mean hydrodynamic diameter (nm) and PDI of mixed polyplexes.

**N/P 10**		**N/P 15**	
**Polyplex**	Diameter (PDI)	**Polyplex**	Diameter (PDI)
**5-10-5G.A**	157 ± 4 (0.23 ± 0.01)	**5-15-5G.A**	149 ± 1 (0.14 ± 0.02)
**5-10-5G.D**	169 ± 5 (0.13 ± 0.02)	**5-15-5G.D**	197 ± 5 (0.33 ± 0.01)
**5-10-5G.AD**	167 ± 5 (0.13 ± 0.01)	**5-15-5G.AD**	196 ± 3 (0.24 ± 0.05)
**5-10-5G.T**	158 ± 4 (0.18 ± 0.01)	**-**	-
**5-10-5G.I**	164 ± 4 (0.22 ± 0.01)	**-**	-
**5-10-5G.TI**	180 ± 3 (0.18 ± 0.01)	**-**	-

Table S10. Mean hydrodynamic diameter (nm) and PDI of polyplexes doped with catechols O, P, and C.

Polyplex	Diameter (PDI)
**5-10-5 G.O**	241 ± 6 (0.26 ± 0.01)
**5-10-5 G.P**	185 ± 5 (0.14 ± 0.02)
**5-10-5 G.C**	233 ± 5 (0.32 ± 0.02)
**5-10-5 G.OPC**	216 ± 3 (0.22 ± 0.03)

pH-Sensitivity of Polyplexes by DLS

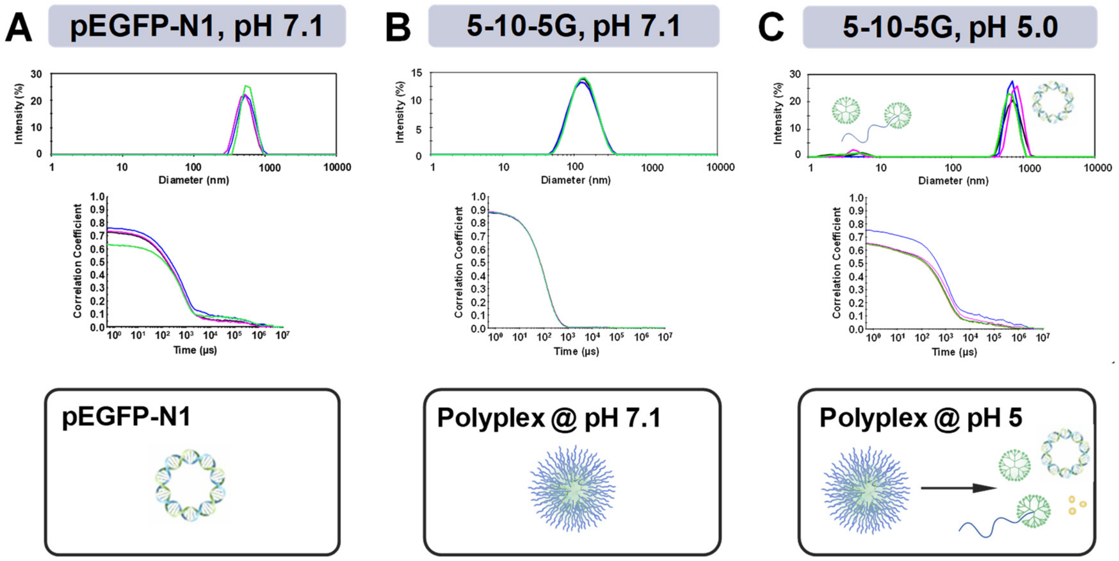

Figure S1. DLS size distributions and correlation functions of pEGFP-N1 (10 ng/µL in 10 mM HEPES 7.1) (**A**), and the polyplex 5-10-5G (10 ng/µL of pEGFP-N1) in 10 mM HEPES 7.1 (**B**) and after acidification at pH 5.0 (0.1 M HCl, 300 rpm orbital stirring in an Eppendorf Thermomixer C for 18 h at 37 ºC (**C**).

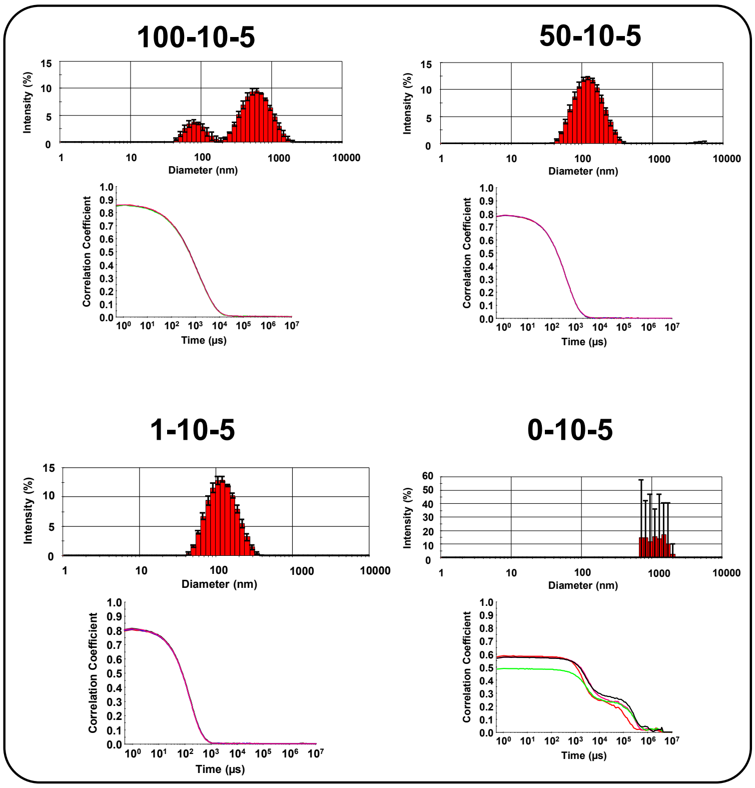

Figure S2. DLS histograms and correlation functions of polyplexes prepared with Cat-G at different %PEG.

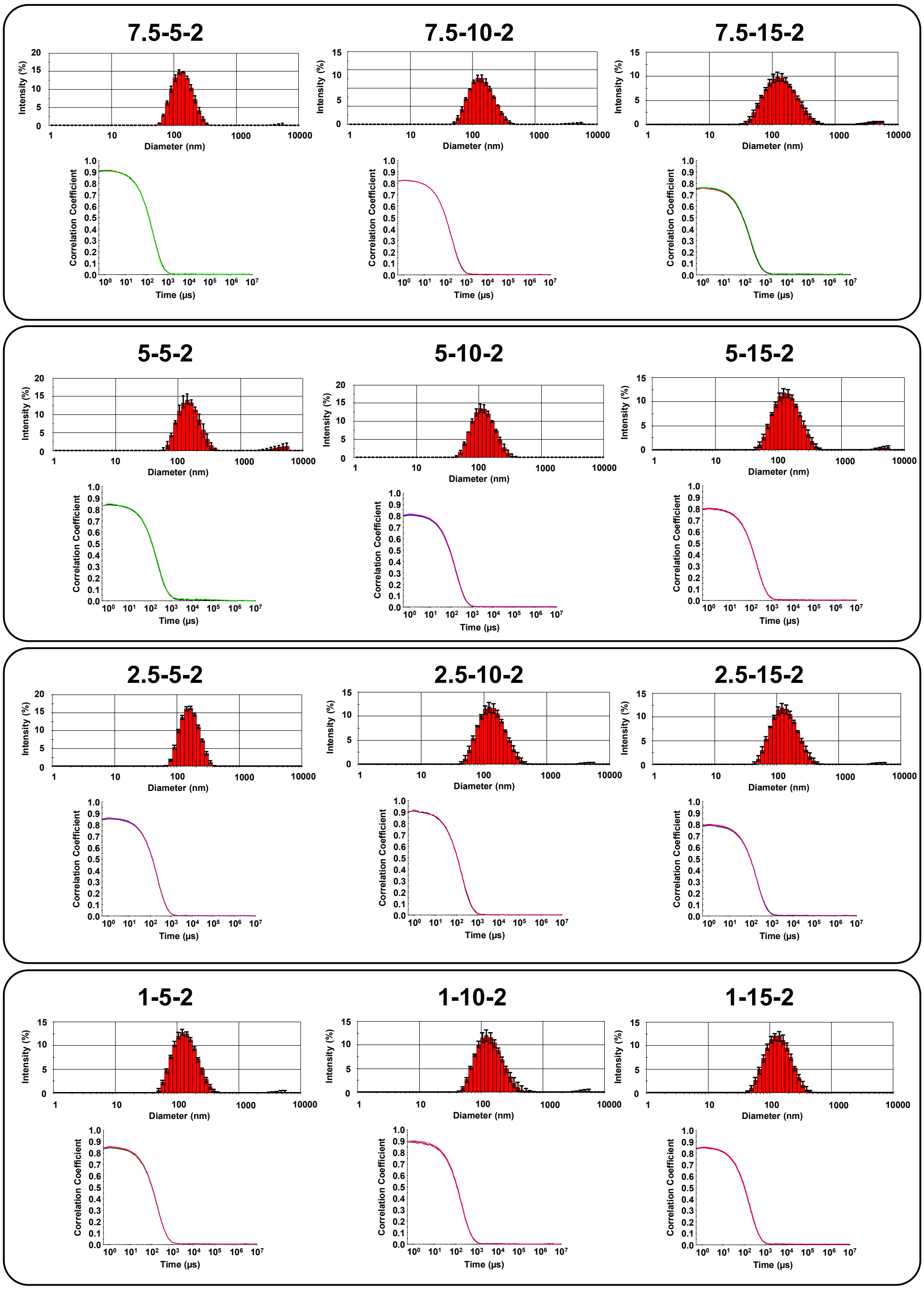

Figure S3. DLS histograms and correlation functions of polyplexes prepared with Cat-G at different N/P and CBA ratios.

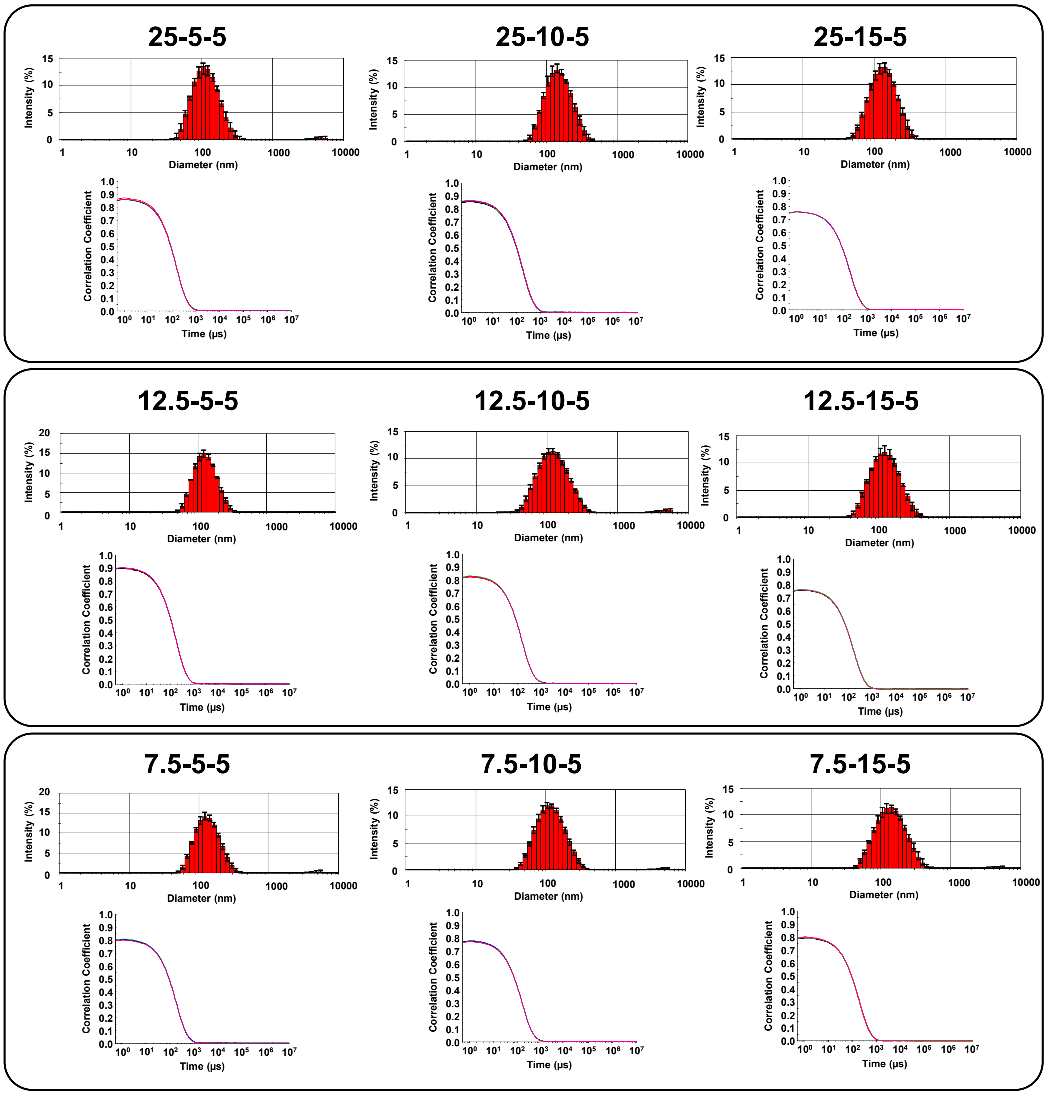

Figure S4. DLS histograms and correlation functions of polyplexes prepared with Cat-G at different N/P and CBA ratios.

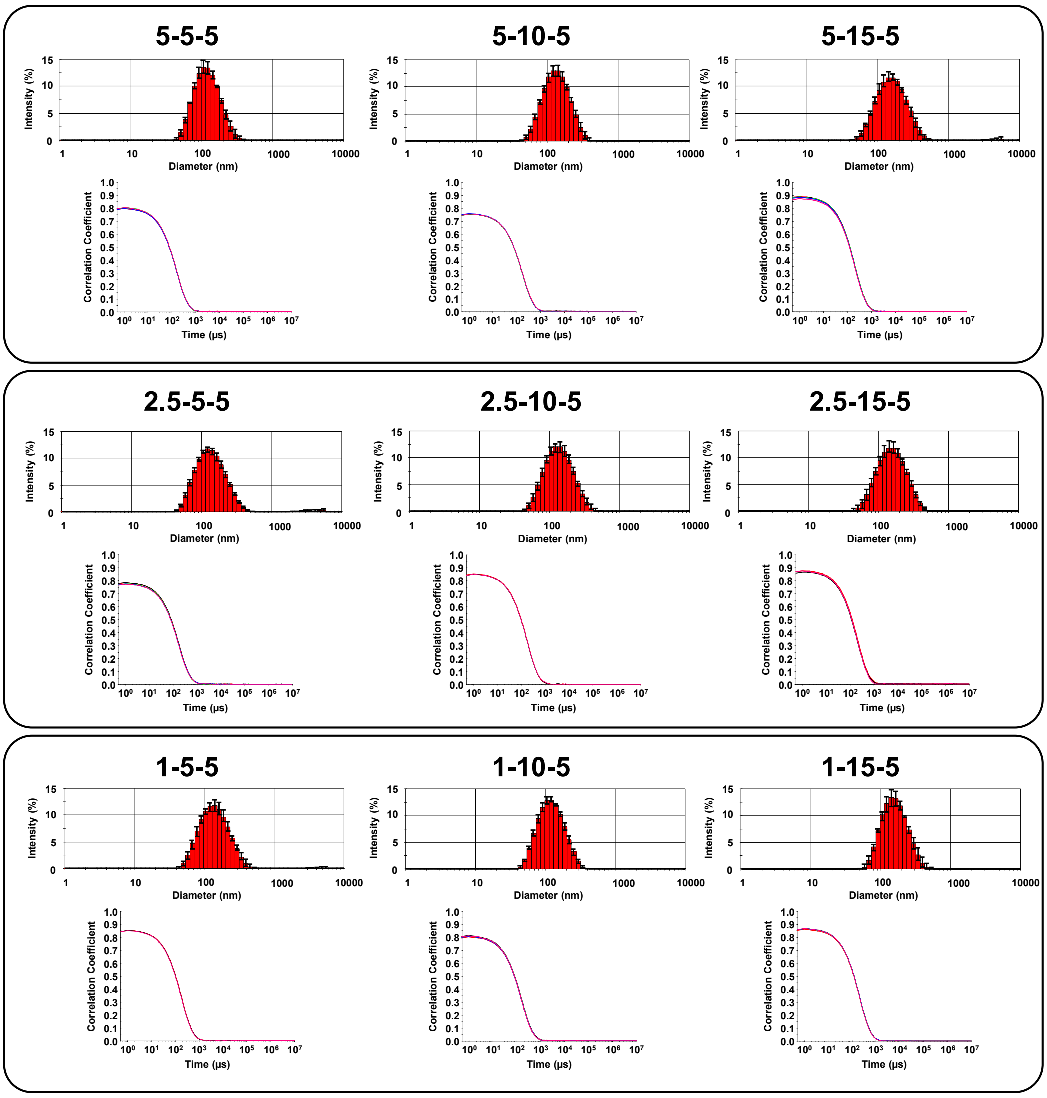

Figure S5. DLS histograms and correlation functions of polyplexes prepared with Cat-G at different N/P and CBA ratios.

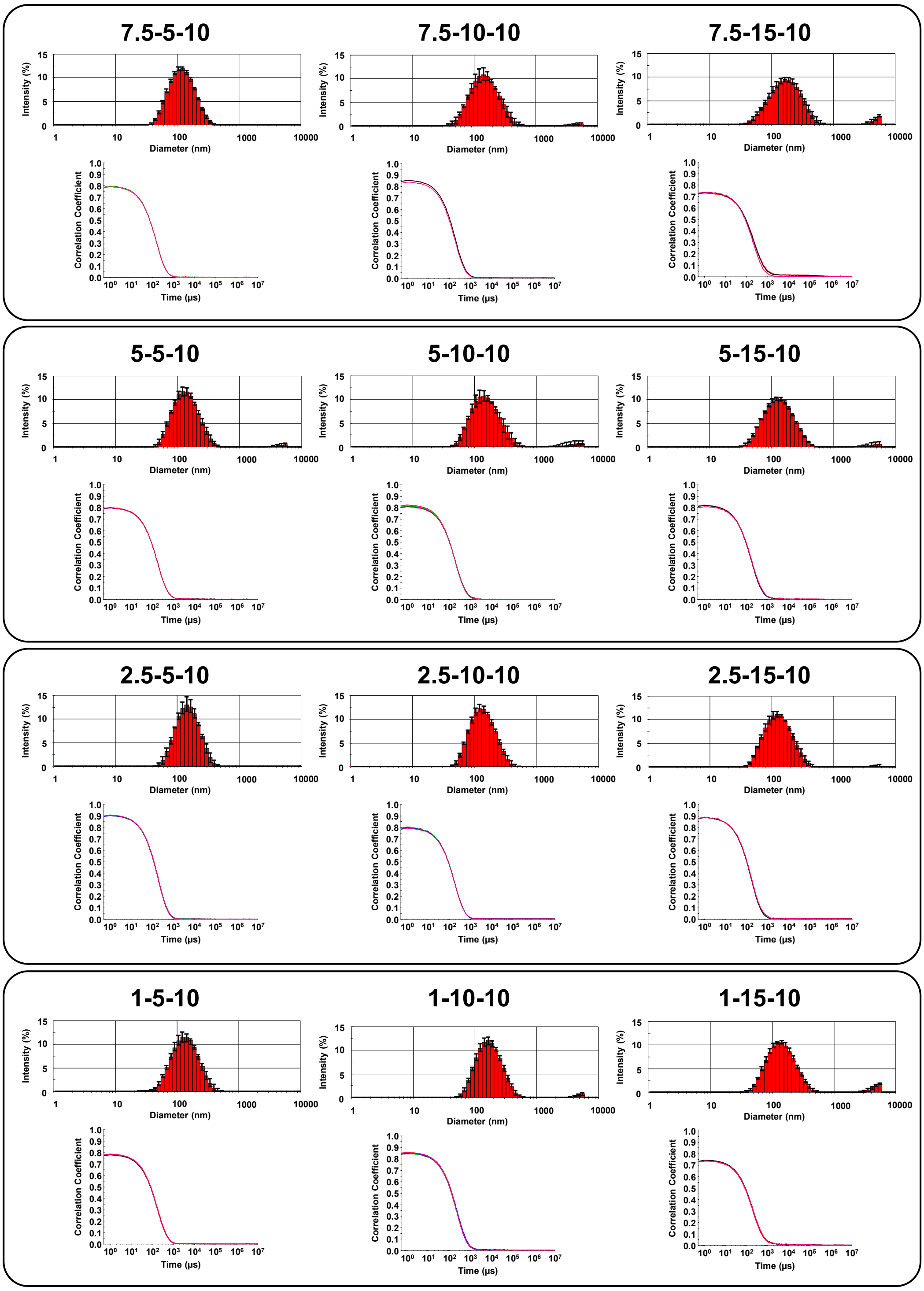

Figure S6. DLS histograms and correlation functions of polyplexes prepared with Cat-G at different N/P and CBA ratios.

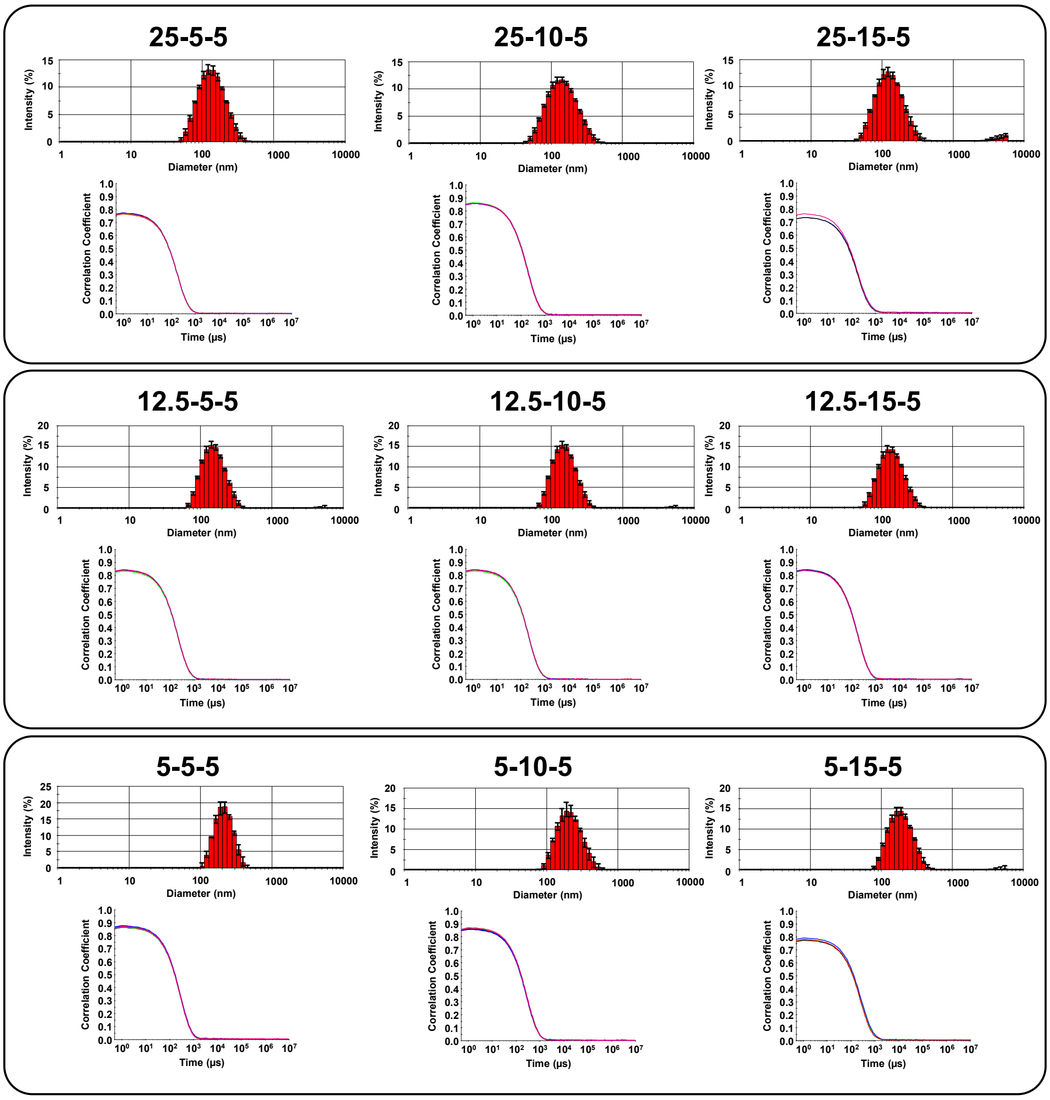

Figure S7. DLS histograms and correlation functions of polyplexes prepared with Cat-A at different N/P.

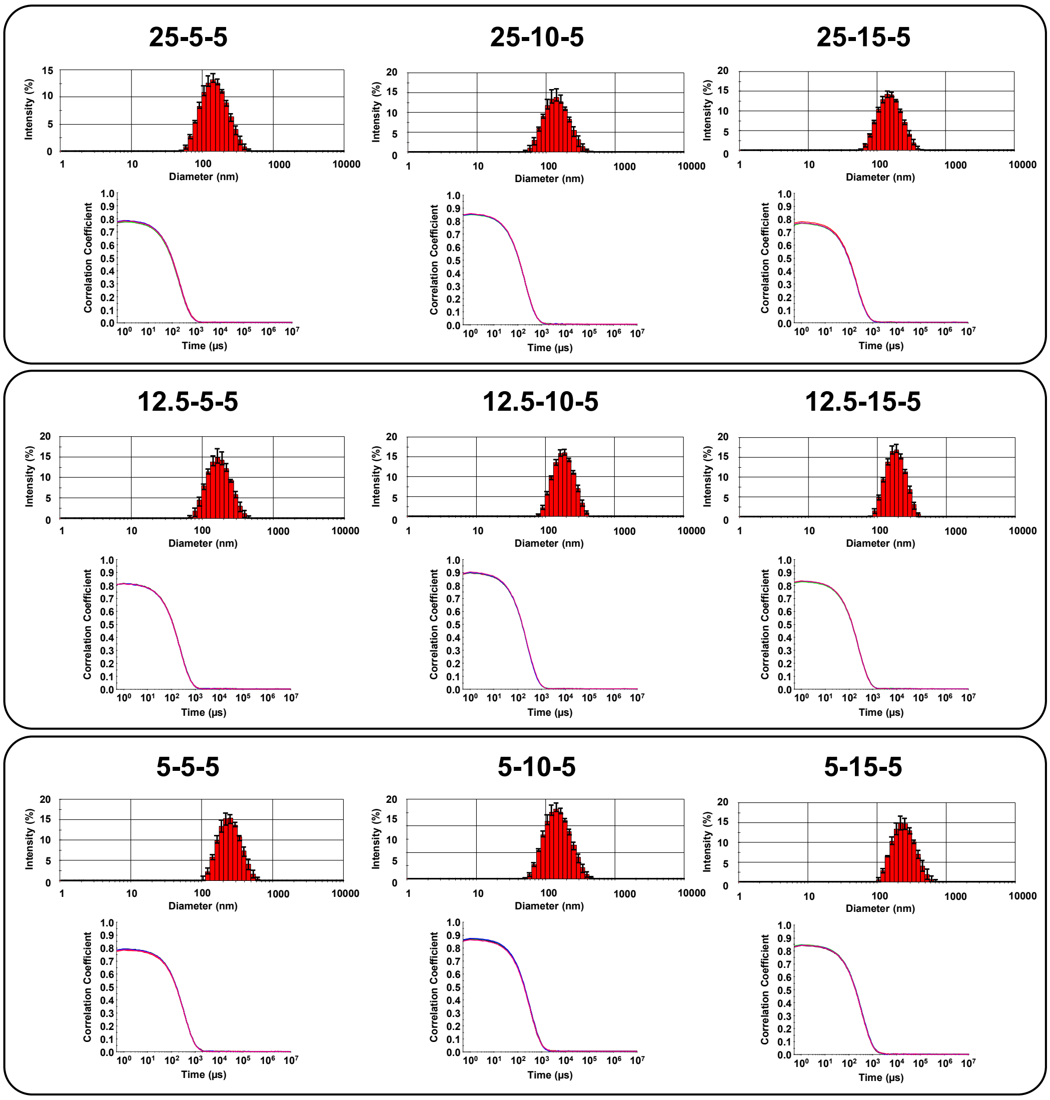

Figure S8. DLS histograms and correlation functions of polyplexes prepared with Cat-D at different N/P.

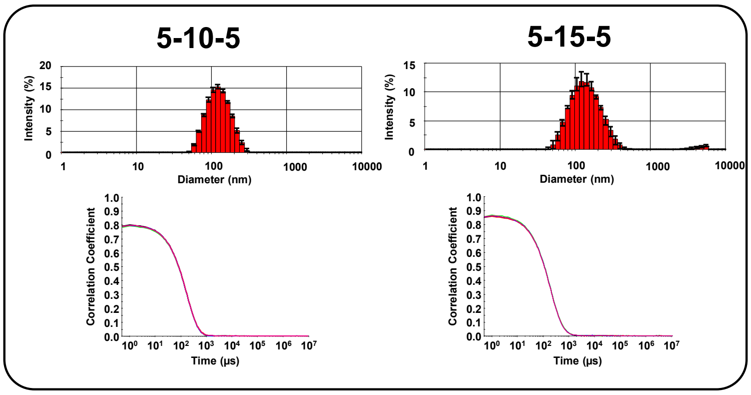

Figure S9. DLS histograms and correlation functions of polyplexes prepared with a mixture of Cat-G (75 %) and Cat-A (25 %) at different N/P.

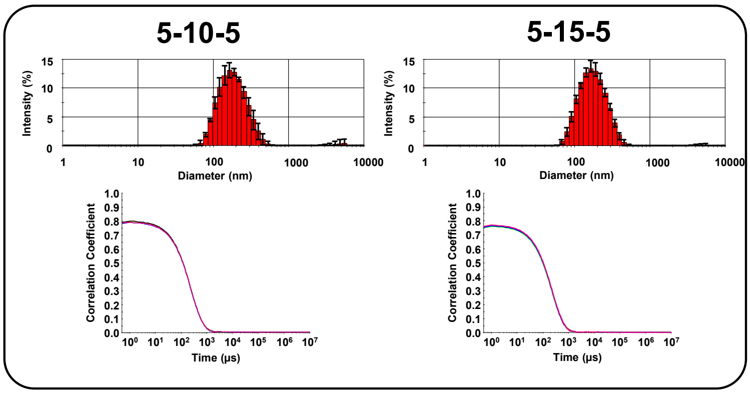

Figure S10. DLS histograms and correlation functions of polyplexes prepared with a mixture of Cat-G (75 %) and Cat-D (25 %) at different N/P.

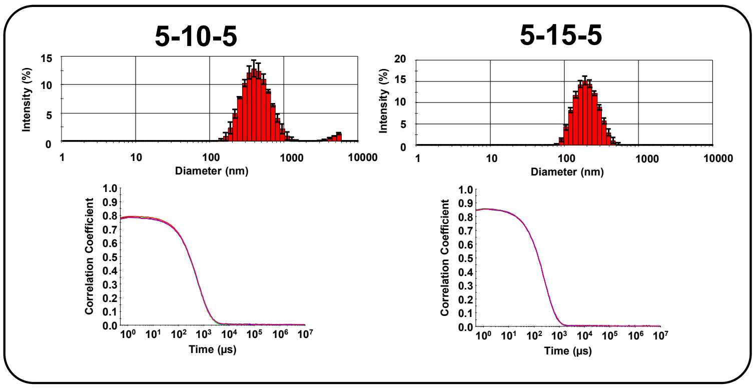

Figure S11. DLS histograms and correlation functions of polyplexes prepared with a mixture of Cat-G (50 %), Cat-A (25 %), and Cat-D (25 %) at different N/P.

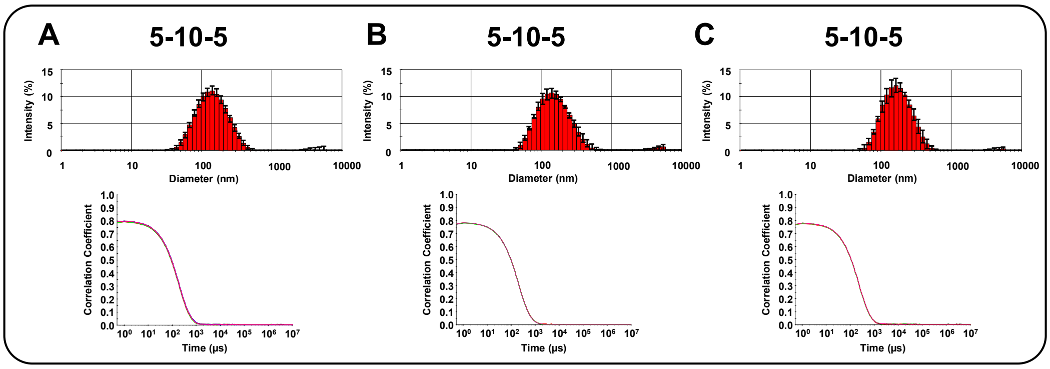

Figure S12. DLS histograms and correlation functions of polyplexes prepared with mixtures of: 75% Cat-G and 25% Cat-T (**A**), 75% Cat-G and 25% Cat-I (**B**), and 50% Cat-G, 25% Cat-T, and 25% Cat-I (**C**).

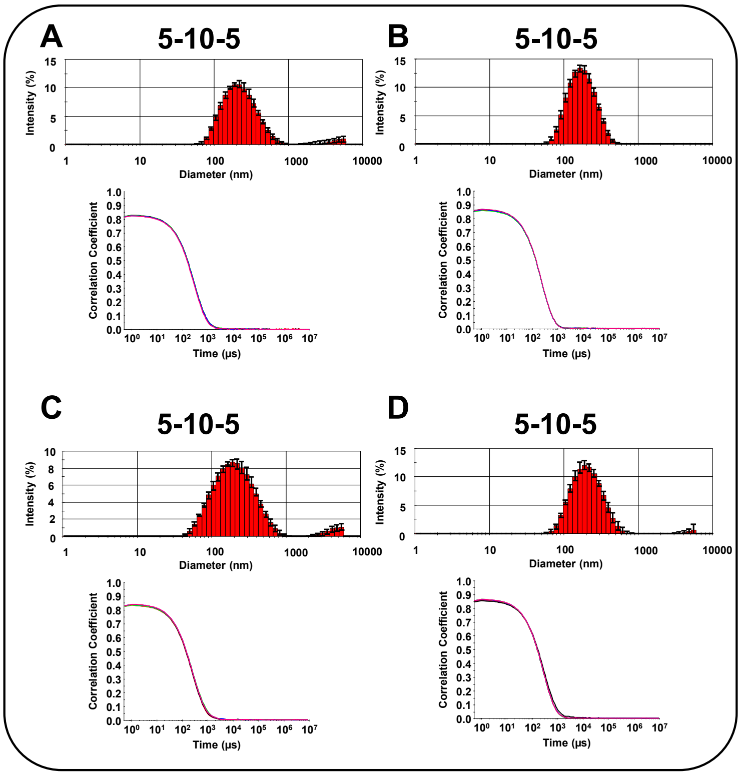

Figure S13. DLS histograms and correlation functions of G polyplexes doped with: 10 mol% Cat-O (**A**), 10 mol% Cat-P (**B**), 10 mol% Cat-C (**C**), and 3.33 mol% each of Cat-O, Cat-P, and Cat-C (**D**).

6. Cell Studies

Cell Cultures. HEK-293, COS-7, HepG2, and HeLa cells, obtained from the European Collection of Authenticated Cell Cultures (ECACC), were cultured at 37 °C in a 5% CO_2 atmosphere in Dulbecco's modified Eagle's medium (DMEM) with high glucose, containing 10% FBS and supplemented with 50 U/mL penicillin and 50 U/mL streptomycin, in 75 cm^2 cell culture flasks. All cell experiments were performed at 37 °C in a 5% CO_2 atmosphere with this modified DMEM, simply referred as "medium".

In vitro Transfection. Cells were seeded in 24-well plates at a density of 200000 cells/mL for HEK-293, 150000 cells/mL for COS-7, 125000 cells/mL for HepG2, and 100000 cells/mL for HeLa. After 24 h of incubation, medium was replaced with fresh medium (250 µL) and then, the polyplex solution was added (1 µg pEGFP-N1/well). Cells were incubated with the polyplex for 5 h. Afterwards, the medium was replaced with fresh medium (500 µL) and incubation continued for up to a total of 48 h (COS-7, HepG2, and HeLa) or 72 h (HEK-293). Transfection with Lipofectamine 2000 (LP 2000) was performed following manufacturer's protocol (FBS-free medium). To verify cell tolerance towards medium replacement, duration of transfection, and washing procedures, controls were conducted by subjecting the cells to the same treatments.

Flow Cytometry. EGFP expression of transfected cells was analyzed by flow cytometry. After 48 h (COS-7, HepG2, and HeLa) or 72 h (HEK-293) of incubation with the polyplexes, cells were washed with PBS (3 × 500 µL) and trypsinized (100 µL of 0.25% Trypsin–EDTA) for 5 min, followed by trypsin quenching with 350 µL of PBS supplemented with 5% FBS. Cells were transferred to 96-well plates and analyzed by flow cytometry collecting at least 5000 events per well using a Guava Millipore flow cytometer equipped with a 488 nm blue laser coupled with a 525/30 nm filter. All reported data represent the average of at least three independent experiments. Data

analysis was performed with InCyte software included in Millipore GuavaSoft 3.2 or with FlowJo. Gating was conducted by performing control experiments in which cells were untreated or treated with different control samples. Results are reported as the percentage of EGFP positive cells and as the median fluorescence intensity of positive cells.

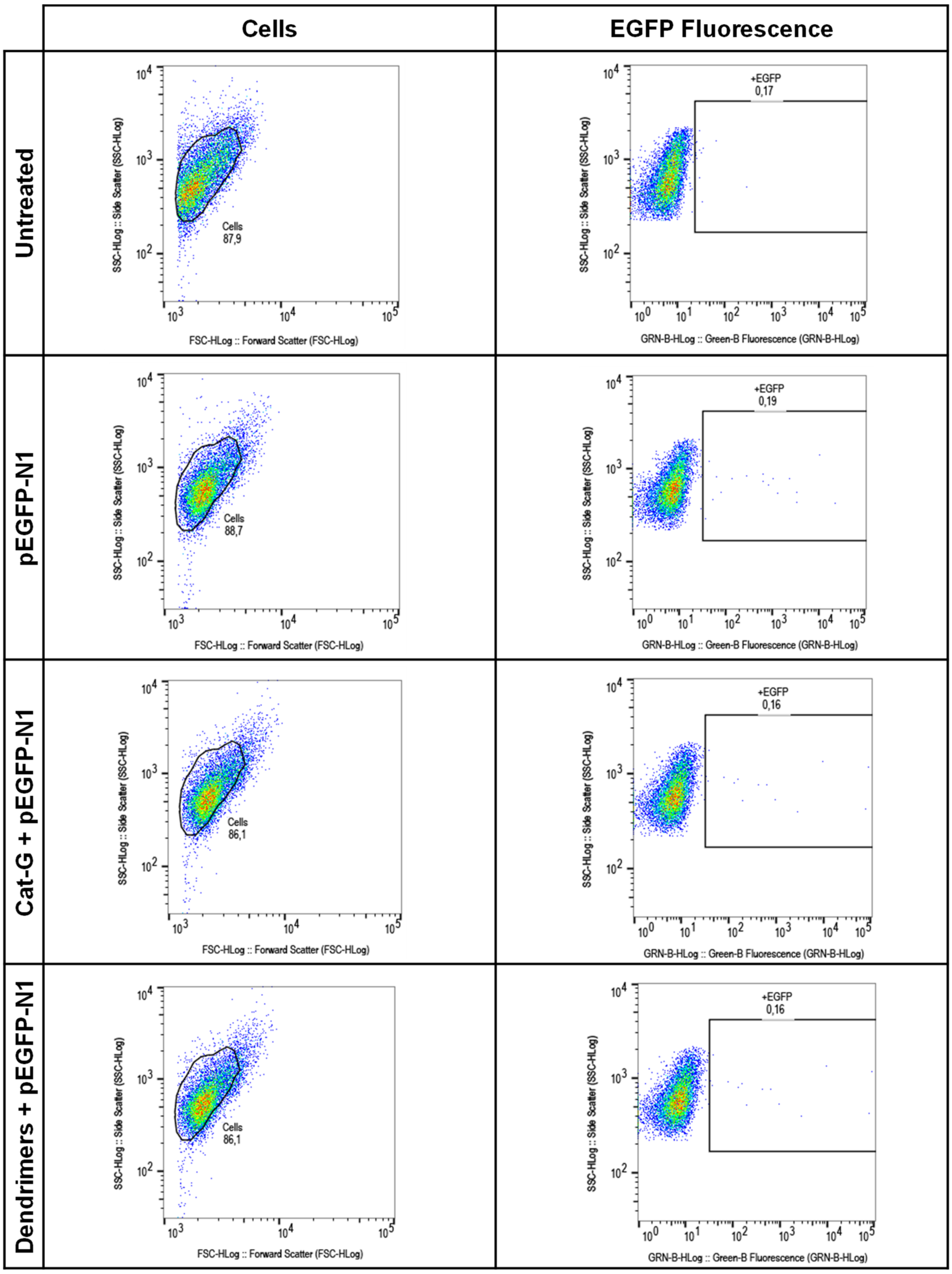

Figure S14. Dot plot displaying SSC *vs* FSC showing the region selected as cells for subsequent analysis (first column). Dot plot displaying SSC *vs* EGFP fluorescence intensity showing the region selected as EGFP positive cells (second column). Experiments performed with (i) untreated HEK-293 cells, and cells after 72 h of incubation with (ii) pEGFP-N1, (iii) a mixture of pEGFP-N1 and Cat-G (equivalent to polyplex 5-10-5G devoid of PEG[G3]-BA and 3[G2]-BA), and a mixture of pEGFP-N1 and dendrimers (equivalent to polyplex 5-10-5G devoid of Cat-G).

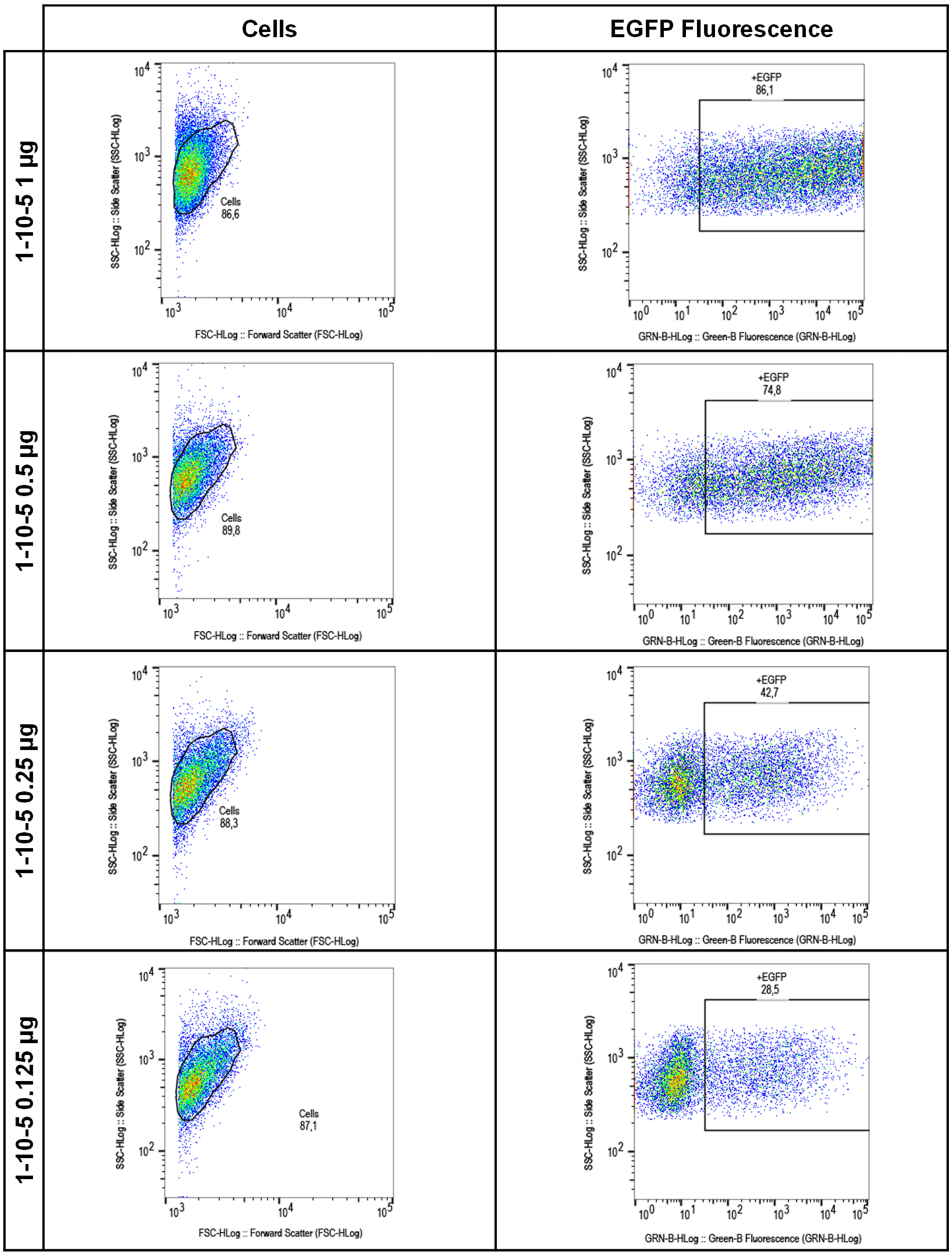

Figure S15. Dot plot displaying SSC *vs* FSC showing the region selected as cells for subsequent analysis (first column). Dot plot displaying SSC *vs* EGFP fluorescence intensity showing the region selected as EGFP positive cells (second column). Dose–dependent transfection of polyplex 1-10-5G after 72 h in HEK-293 cells.

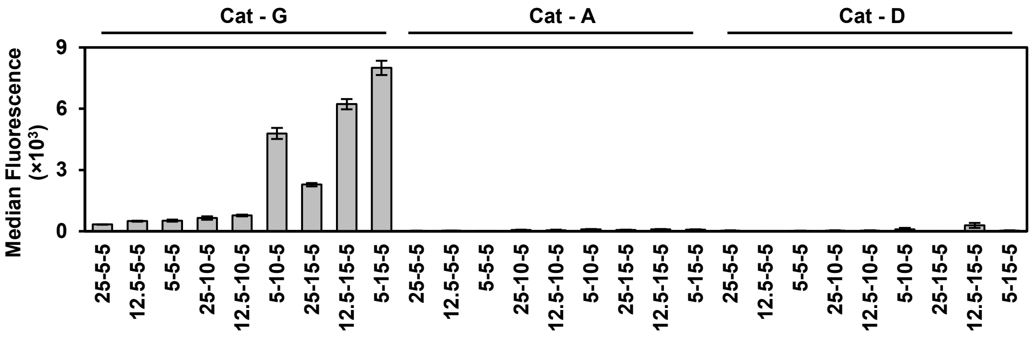

Figure S16. Median EGFP fluorescence of HEK-293 cells transfected for 72 h with G-, A-, and D-polyplexes of different %PEG and N/P ratios (CBA 5) measured by flow cytometry.

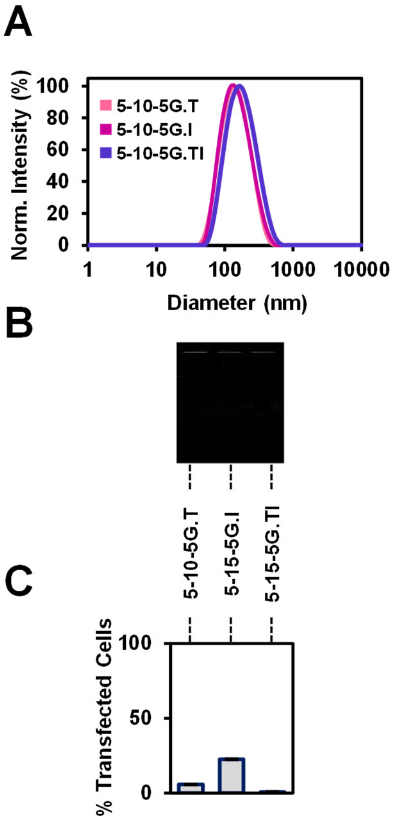

Figure S17. Polyplex 5-10-5G doped with Cat-T and Cat-I. DLS histograms (**A**), gel retardation experiments with ethidium bromide on agarose gel electrophoresis (**B**), and percentage of transfected HEK-293 cells after 72 h of incubation (**C**).

Cell Viability. After 72 h of transfection, cell viability was determined by a colorimetric assay with CCK-8 following manufacturer's protocol. After 2 h of incubation at 37 °C in 5% CO_2 with a 6% solution of CCK-8 in medium, supernatant solution was transferred to a 96-well plate. Viability was determined by measuring the supernatant absorbance at 450 nm in a plate reader Tecan Infinite F200 PRO. Absorbance (*A*) from a 6% CCK-8 solution in medium was subtracted from all data points. Viability was calculated as follows:

$$\text{Cell Viability (\%)} = 100 \times \frac{(A_{\text{sample}} - A_{\text{6\% CCK-8}})}{(A_{\text{control}} - A_{\text{6\% CCK-8}})}$$

Fluorescence Microscopy. HEK-293 cells were seeded in 24-well glass bottom 15 mm plates (Cellvis) at a density of 200000 cells/mL in 500 μL of medium. After 24 h of incubation at 37 °C in 5% CO_2, culture media was replaced with 250 μL of medium and polyplex solution (1 μg pEGFP-N1/well). Cells were then incubated at 37 °C in 5% CO_2 for 5 h, followed by a replacement of culture media with fresh medium and a new incubation for 72 h at 37 °C in 5% CO_2. Then, EGFP expression was detected by fluorescence microscopy using a Nikon TiE microscope equipped with a Zyla 4.2 PLUS camera (Andor, Oxford Instruments). Excitation 475/35 nm; emission 530/43 nm.

Internalization Studies by Laser Scanning Confocal Microscopy (LSCM). HEK-293 were seeded in 24-well glass bottom 15 mm plates (Cellvis) at a density of 200000 cells/mL in 500 μL of medium. After 24 h of incubation at 37 °C in 5% CO_2, culture media was replaced with 250 μL of medium and polyplex solution (1 μg pEGFP-N1-Cy5/well). Cells were then incubated at 37 °C in 5% CO_2 for 5 h. Half an hour before observation, acidic organelles were stained by adding 10 μL of LysoTracker Red (10 μM in 10% DMSO:H_2O) and nuclei were stained by adding 10 μL of Hoechst 33258

(200 μM in 10% DMSO:H_2O). Before observation, culture media was removed, cells were washed with DMEM and 500 μL of fresh medium was added.

Confocal images were obtained on an Andor Dragonfly spinning disk confocal system mounted on a Nikon TiE microscope equipped with a Zyla 4.2 PLUS sCMOS digital camera (Andor, Oxford Instruments) and an OKO-lab incubator to maintain cells at 37 °C. Samples were excited with three different lasers (405, 561, and 637 nm) and the emitted fluorescence was collected by the filter wheel (450/50 nm, 620/50 nm, and 725/40 nm) with appropriate combinations of them.

Internalization Studies by Flow Cytometry. HEK-293 cells were seeded in a 24-well plate at a density of 200000 cells/mL. After 24 h of incubation at 37 °C in 5% CO_2, medium was replaced with 250 μL of medium and polyplex solution (1 μg pEGFP-N1-Cy5/well). After 5 h of incubation at 37 °C in 5% CO_2, cells were washed with PBS (3 × 500 μL) and trypsinized (100 μL 0.25% Trypsin–EDTA) for 5 min followed by trypsin quenching with 350 μL of serum-supplemented PBS. Then, cells were pelleted by centrifugation (5 min, 700 *g*), washed with 500 μL of serum-supplemented PBS, and pelleted again using the same conditions. Finally, cells were resuspended with 500 μL of serum-supplemented PBS and the suspension was transferred into FACS tubes for analysis by flow cytometry. At least 10000 events were collected using a Becton-Dickinson FACSCalibur Flow Cytometer equipped with a 635 nm red laser coupled with a BP 660/13 nm filter. For the analysis, cells population was selected by representing FSC *vs* SSC. Then, histograms displaying the number of events (count) *vs* Cy5 fluorescence were obtained. The region of Cy5 positive cells was delimited referring to untreated cells (control). Results are reported as the percentage of Cy5 positive cells.

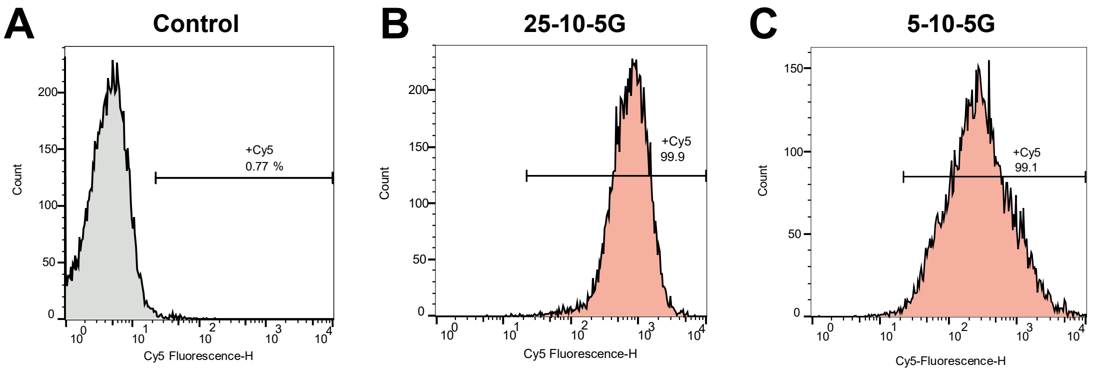

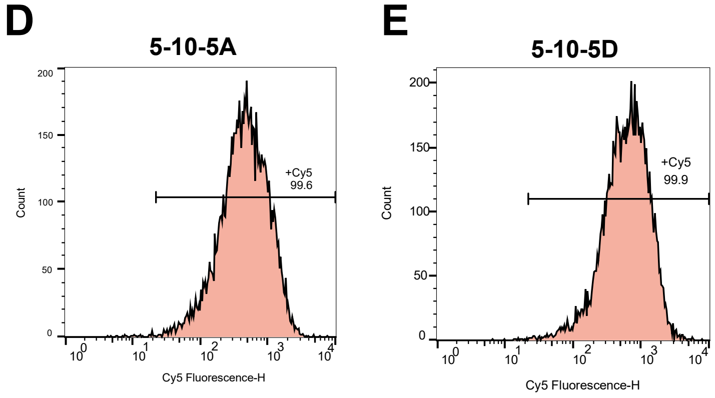

F

Polyplex	%Cells	% +Cy5
**25-10-5G**	79	99.9
**5-10-5G**	70.3	99.1
**5-10-5A**	76.2	99.6
**5-10-5D**	77.3	99.9
**Control**	74.2	0,77

Figure S18. Histograms displaying number of cells (count) *vs* Cy5 fluorescence intensity showing the region selected as Cy5 positive cells. Experiments performed with untreated HEK-293 cells (**A**), and cells after 5 h of incubation with 25-10-5G (**B**), 5-10-5G (**C**), 5-10-5A (**D**) and 5-10-5D (**E**). Results are reported as the percentage of Cy5 positive cells (**F**).

Erythrocyte Aggregation Assay. Fresh rat blood was collected and immediately mixed with sodium citrate to a final concentration of 25 mM. Erythrocytes were obtained after multiple rounds of centrifugation in cold PBS, until the supernatant became clear (1600 *g* at 4 °C for 10 min). A 3% (w/v) suspension of erythrocytes was then prepared in PBS. Aliquots of an erythrocyte suspension (100 µL, 1% w/v in PBS) were mixed with 100 µL of solutions with increasing concentrations of PEG[G3]-BA or 3[G2]-BA and Cat-G at a CBA ratio of 5 in PBS (final concentrations: 25, 50, 100, and 250 µg/mL). The resulting suspensions were transferred to 24-well glass bottom 15 mm plates (Cellvis) and incubated at 37 °C in 5% CO_2. After 1 h of incubation, the degree of erythrocyte

aggregation was analyzed by imaging the samples using a Nikon TiE microscope equipped with a Zyla 4.2 PLUS camera (Andor, Oxford Instruments).

Hemolysis Assay. Aliquots of an erythrocyte suspension (100 µL, 3% w/v in PBS) were mixed with 100 µL of solutions with increasing concentrations of PEG[G3]-BA or 3[G2]-BA and Cat-G at a CBA ratio of 5 in PBS (final concentrations: 5, 25, 50, 100, and 250 µg/mL). The resulting suspensions were incubated for 1 h at 37 °C under orbital shaking. Then, cells were removed by centrifugation (1600 *g*, 10 min) and hemoglobin release was analyzed by measuring the supernatant absorbance (A) at 540 nm. PBS and 2% Triton X-100 were used as negative (non-hemolytic, 0% hemoglobin release) and positive (hemolytic, 100% hemoglobin release) controls, respectively. Absorbance measurements were performed in a plate reader Tecan Infinite F200 PRO. The percentage of hemolysis was calculated as follows:

$$\text{Hemolysis (\%)} = 100 \times \frac{(A_{\text{sample}} - A_{\text{control}})}{(A_{\text{2\% Triton X-100}} - A_{\text{control}})}$$

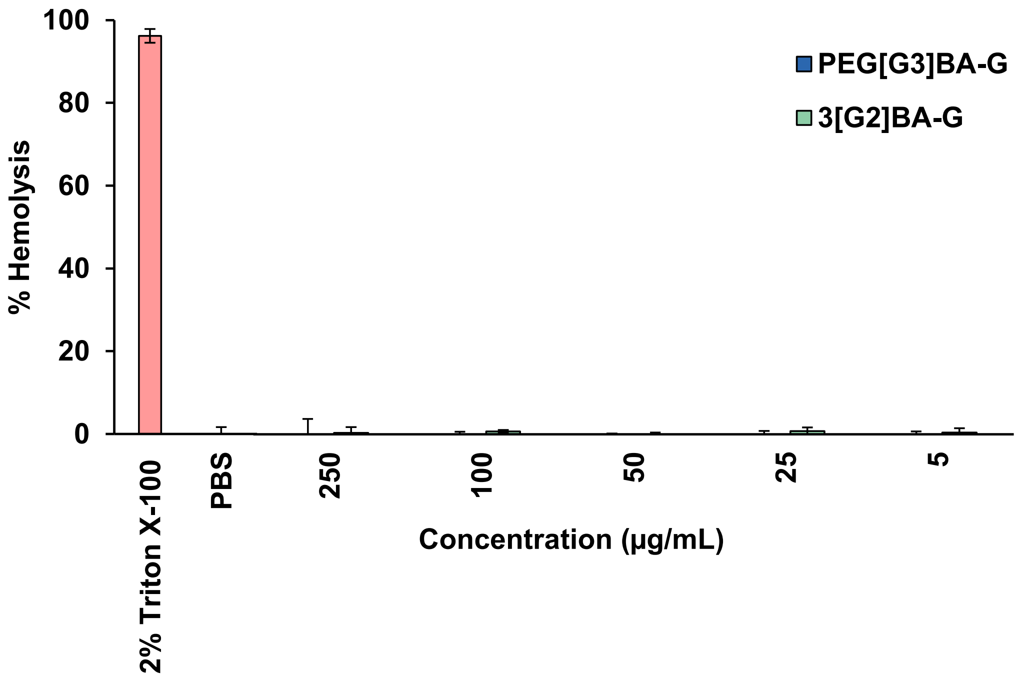

Figure S19. Percentage of hemoglobin released upon incubation of erythrocytes (3% w/v in PBS) with increasing concentrations of PEG[G3]-BA or 3[G2]-BA and Cat-G at a CBA ratio of 5, for 1 h at 37 °C. The amount of hemoglobin released was determined by absorbance of the supernatant (540 nm), using PBS and 2% Triton X-100 as negative and positive controls, respectively.

7. *In vitro* Assays

Protection against decomplexation. The ability of cationic polyboronate vectors to protect pEGFP-N1 against decomplexation by glycosaminoglycans/polyanions was assessed in 96-well plates by monitoring the fluorescence increase of ethidium bromide permeating the polyplex and intercalating into pDNA. Untreated polyplexes (no fluorescence) and naked pDNA (maximum fluorescence, complete ethidium bromide accessibility to pDNA) were employed as negative and positive controls, respectively.

Briefly, polyplexes 1-10-5G and 1-15-5G (0.6 µg of pEGFP-N1, 8 µL per well) were incubated for 2 h at 37 ºC under horizontal shaking with increasing concentrations of hyaluronic acid, heparin, and dextran sulfate (92 µL, PBS), which accounted for up to a

18 charge ratio relative to the negative phosphate groups of pEGFP-N1 (equivalent to 138 µg/mL of hyaluronic acid, 55 µg/mL of heparin, and 43 µg/mL of dextran sulfate). Once at room temperature, ethidium bromide (0.5 µg) was added to each well and its fluorescence measured in a plate reader Tecan Infinite F200 PRO (λ_{ex} 300 nm, λ_{em} 600 nm).

DNase I Protection Assay. DNase I (1 U DNase/µg pEGFP-N1) was added over 100 µL of polyplexes 1-10-5G and 1-15-5G (10 ng of pEGFP-N1/µL) in 10 mM HEPES 7.1. After incubation for 60 min at 37 °C, samples were cooled at 0 °C and the enzymatic reaction was quenched by adding 5 µL of 0.5 M EDTA. Complexed pEGFP-N1 was partially dissociated from polyplexes by treatment with dextran sulfate (4 µL of a 50 mg/mL solution in 10 mM HEPES 7.1) for 60 min at 37 °C. The topology of the released pEGFP-N1 was examined by 0.7% agarose gel electrophoresis using naked pEGFP-N1 as control.

8. References

1. Zhao, A. Y.; Brooks, A. F.; Raffel, D. M.; Stauff, J.; Arteaga, J.; Scott, P. J. H.; Shao, X. Fully Automated Radiosynthesis of [^{11}C]Guanidines for Cardiac PET Imaging. *ACS Med. Chem. Lett.* **2020**, *11*, 2325-2330.

2. Amato, A.; Migneco, L. M.; Martinelli, A.; Pietrelli, L.; Piozzi, A.; Francolini, I. Antimicrobial activity of catechol functionalized-chitosan versus Staphylococcus epidermidis. *Carbohydr. Polym.* **2018**, *179*, 273-281.

3. Fernandez-Villamarin, M.; Sousa-Herves, A.; Correa, J.; Munoz, E. M.; Taboada, P.; Riguera, R.; Fernandez-Megia, E. The Effect of PEGylation on Multivalent Binding: A Surface Plasmon Resonance and Isothermal Titration Calorimetry Study with Structurally Diverse PEG-Dendritic GATG Copolymers. *ChemNanoMat* **2016**, *2*, 437-446.

4. Amaral, S. P.; Tawara, M. H.; Fernandez-Villamarin, M.; Borrajo, E.; Martínez-Costas, J.; Vidal, A.; Riguera, R.; Fernandez-Megia, E. Tuning the Size of Nanoassembles: A Hierarchical Transfer of Information from Dendrimers to Polyion Complexes. *Angew. Chem. Int. Ed.* **2018**, *57*, 5273-5277.
